# Immune checkpoint inhibitor therapy in advanced cancer: clinical association of irAEs type, inflammatory markers and efficacy

**DOI:** 10.3389/fimmu.2025.1662333

**Published:** 2025-11-26

**Authors:** Mengying Qian, Ping Ma, Yu Zhao, Hao Jiang, LiYang Gao, Gaoyang Lin, Difan Duan, Jinmin Guo

**Affiliations:** 1 School of Pharmacy, Shandong Second Medical University, Weifang, Shandong, China; 2 Department of Pharmacy, 960th Hospital Joint Logistics Support Force of the People Liberation Army, Jinan, Shandong, China; 3 Affiliated Cancer Hospital of Shandong First Medical University, Jinan, China; 4 School of Pharmacy, Jinzhou Medical University, Jinzhou, China; 5 Jinan Key Laboratory of Individualized Clinical Drug Safety Monitoring and Pharmacovigilance Research, Shandong, Jinan, China

**Keywords:** immune checkpoint inhibitors, inflammatory biomarkers, immune-related adverse events, overall survival, meta-analysis

## Abstract

**Background:**

Immune checkpoint inhibitors (ICIs) improve survival in advanced cancers but are associated with immune-related adverse events (irAEs), whose prognostic impact remains debated. The role of systemic inflammatory biomarkers is also not fully defined.

**Methods:**

This research merged a comprehensive systematic review and meta-analysis of 38 studies involving 55,966 participants with a multicenter retrospective cohort study of 870 patients receiving ICI therapy. The aim of this study was to examine the association between irAE characteristics and severity, and baseline inflammatory indicators (NLR, dNLR and PLR), with clinical outcomes, particularly survival (OS) and disease progression survival (PFS). The data were analyzed through time-dependent cox model and meta-analysis.

**Results:**

Among 870 immunotherapy patients, 32.4% developed irAEs, predominantly grade 1-2 (83.9%). Severe irAEs (grade >2) significantly increased mortality (OS HR = 1.93). Organ-specific analysis identified endocrine (HR = 0.938, p < 0.001) and skin toxicity (HR = 0.763, p<0.001) as independent protective factors for OS, while hepatic (HR = 1.602, p=0.031) and cardiac toxicity (HR = 1.181, p=0.017) were risk factors. Elevated baseline inflammatory markers—MLR >0.47 (HR = 3.37), NLR >3.45 (HR = 2.24), and PLR >186.98 (HR = 2.10)—also predicted poorer OS. A meta-analysis confirmed that low-grade irAEs (grade ≤2) conferred significant survival benefit (OS HR = 0.54), particularly skin and endocrine toxicities. These findings support irAEs as biomarkers of immunotherapy response, with prognostic relevance shaped by severity and organ involvement.

**Conclusions:**

The prognosis of irAEs depends on organ involvement and severity. Endocrine and skin toxicities confer survival benefits, while severe, hepatic, and cardiac events pose significant risks. Inflammatory markers predict survival but not irAE onset.

## Introduction

Over the past decade, immune checkpoint inhibitors (ICIs) have significantly enhanced outcomes and increased survival for patients with various advanced solid tumors through blocking key immune regulators such as PD-1/PD-L1 and CTLA-4 (1, 2). The finding has had a significant impact on the fields of cancer treatment. Although ICIs increase anti-tumor immunity, they can also disrupt immune tolerance, which can lead to immune-related adverse events (irAEs). According to clinical evidence, patients receiving anti-CTLA-4 therapy may experience up to 90% of irAEs, while patients getting anti-PD-1/PD-L1 treatment had a cumulative frequency of 70% (3). The integumentary, gastrointestinal, respiratory, endocrine, and musculoskeletal systems are just a few of the organ systems in the human body that these irAEs might impact (4–6). Certain adverse effects may endure over time or emerge at a delayed stage, potentially resulting in a chronic disease trajectory (6).

One of the main areas of current research is the link between irAEs and clinical outcomes. According to the widely accepted theory, better clinical results may be associated with the incidence of irAEs. However, existing evidence indicates that this association is not universally applicable, exhibiting considerable heterogeneity across various studies and patient populations. Notably, irAEs affecting the endocrine system (7–9), such as thyroid dysfunction, and cutaneous irAEs demonstrate a higher incidence and a relatively consistent association with favorable prognoses (10). Conversely, certain irAEs, including immune checkpoint inhibitor-associated myocarditis (11) and severe pneumonia (12, 13), are linked to poorer prognoses, treatment interruptions, or diminished therapeutic efficacy. Moreover, the majority of studies have insufficiently accounted for the influence of toxicity severity within a single organ system in their association analyses. Recent investigations have additionally demonstrated that the long-term management of chronic irAEs is significantly correlated with clinical outcomes, indicating that these effects warrant thorough evaluation alongside the characteristics of disease progression (6). Mechanistically, the emergence of irAEs is hypothesized to be related to the induction of, or concurrent systemic, immune-inflammatory states. Importantly, specific peripheral blood markers that reflect the intensity of cancer-associated inflammation, such as the platelet/lymphocyte ratio (PLR), monocyte/lymphocyte ratio (MLR), and neutrophil/lymphocyte ratio (NLR), have been identified as independent predictors of the efficacy of ICIs monotherapy in solid tumors (14, 15). Nevertheless, the predictive value of these inflammatory markers for irAEs and their relationship with treatment efficacy remain inadequately explored and inconclusive. Current research predominantly focuses on cohorts receiving ICI monotherapy. Therefore, the predictive value of these inflammatory markers, along with their intricate associations with irAEs and treatment efficacy in advanced cancer patient populations undergoing ICI therapy, necessitates validation through more comprehensive and extensive large-scale studies.

Consequently, the clinical findings highlight the importance of conducting a thorough examination of the link between irAEs and inflammatory markers in relation to treatment efficacy. This study employed a complementary research design comprising two components: (1) a multicenter retrospective cohort study (N = 870) dynamically tracked the types, onset times, and severity of irAEs, exploring their associations with inflammatory markers, and use the time-dependent models to assess their impact on survival outcomes; and (2) a meta-analysis that integrated individual patient data from 38 studies (n=55,966) to quantify the moderating effects of factors such as tumor type and irAE occurrence through meta-regression and subgroup analysis. This dual validation framework not only enhances statistical efficacy but also elucidates the underlying factors driving the observed associations at the level of clinical characterization.

## Methodology

### Clinical cohort

#### Study design

This two-center retrospective cohort study aims to determine irAEs and their impact on outcomes related to survival in patients undergoing ICI treatment. Clinical data was gathered from the Chinese People’s Liberation Army’s 960th Hospital’s computerized medical record systems and the First Medical University of Shandong, China’s Affiliated Cancer Hospital. Patients who met the inclusion criteria between January 2019 and February 2025 were included in the study. The study embraced the principles of the Declaration of Helsinki and was authorized by the hospital’s ethics committee (approval number 2025–033 and SDTHEC20250748). Given the study’s retrospective methodology, informed consent was not obtained.

#### Eligibility criteria

Participants in this study had to be eligible if they fulfilled the following criteria: (1) received ICIs; (2) received the first dose of ICI between January 2019 and October 2024; and (3) at least 18 years of age. Up until February 2025, the patients were observed. The following were the exclusion criteria: (1) fewer than two therapy cycles; (2) multiple primary tumors; (3) surgery; (4) clinical trial participation; (5) insufficient baseline or follow-up data; and (6) autoimmune disease diagnosis. In the end, 870 patients were found to fit the predetermined requirements.

#### Data collection

Patient baseline characteristics were obtained from the hospital’s electronic medical records. Collected variables comprised demographic and clinical details such as sex, age, alcohol and tobacco use history, cancer diagnosis, Eastern Cooperative Oncology Group (ECOG) performance status, inflammatory indices (e.g., MLR, NLR, PLR, derived neutrophil-lymphocyte ratio [dNLR], systemic inflammation response index [SIRI], systemic immune-inflammation index [SII]), adverse event occurrences, therapeutic regimens, and treatment outcomes. The cutoff thresholds for inflammatory indicators were established through receiver operating characteristic (ROC) curve analysis. To rigorously assess anti-tumor treatment response, participants underwent radiographic imaging (CT or MRI) every two or three cycles, alongside continuous tracking of serum biomarker levels during each treatment cycle. Progression-free survival (PFS) and overall survival (OS) served as the study’s dual primary endpoints. OS meant the time between starting ICI therapy and the patient dying from any reasons and PFS meant the time between starting treatment and the first tumor growing or the patient dying, based on the Response Evaluation Criteria in Solid Tumors (RECIST) version 1.1 (16). Additionally, all irAEs were categorized and graded using the National Cancer Institute’s (NCI) Common Terminology Criteria for Adverse Events version 5.0 (CTCAEv5.0) (17). The occurrence of irAEs was observed from the commencement of immune-related therapy until they became apparent.

#### Statistical analysis

This study provides a systematic description and analysis of the baseline clinical characteristics of the enrolled patient cohort. Continuous variables are expressed as mean ± standard deviation (Mean ± SD), whereas categorical variables are summarized by frequency and percentage. Comparative analyses between groups were conducted using statistical methods appropriate to the variable type and distribution: categorical variables were evaluated via chi-square tests or Fisher’s exact tests, and continuous variables were examined using independent samples t-tests.

To rigorously control for potential confounding factors affecting the analysis of irAEs, a stratified propensity score matching approach was employed instead of uniform matching across the entire sample. Specifically, when comparing different irAE subtypes with the irAE-free control group, as well as within cancer subtype subgroups, irAE status or cancer type was treated as the dependent variable. Covariates incorporated into the matching process included age, sex, coronary heart disease, diabetes, hypertension, smoking status, alcohol consumption, Eastern Cooperative Oncology Group (ECOG) performance status, metastatic sites, number of prior treatment lines, and chemotherapy exposure. Nearest-neighbor matching was performed at a 1:2 ratio, with a caliper width set at 0.2 times the standard deviation of the propensity score. Standardized mean differences (SMDs) for each covariate were calculated before and after matching, with an SMD threshold of less than 0.2 indicating adequate covariate balance. This procedure yielded independent matched cohorts suitable for subsequent subgroup analyses.

In the matched cohorts, survival differences between groups were evaluated using the Kaplan-Meier method in conjunction with the log-rank test. To mitigate potential immortal time bias in the analysis of irAEs, a time-dependent Cox proportional hazards model was employed. This approach facilitated precise estimation of adjusted hazard ratios for irAEs while accounting for multiple clinical confounding variables.

Moreover, the study investigated the prognostic influence of various cancer types and irAE subtypes through stratified analyses. Logistic regression models were also utilized to examine the relationship between baseline inflammatory biomarkers and the risk of irAE development. All statistical analyses were performed using R version 4.4.2 and SPSS version 26.0, with a significance threshold set at α = 0.05.

### Meta-analysis

#### Search strategy

This study adheres to the guidelines provided by the Cochrane Handbook and the Preferred Reporting Items for Systematic Reviews and Meta-Analyses (PRISMA) (18). To find papers that satisfied the qualifying requirements, a complete search of pertinent databases, including PubMed, Cochrane Library, Web of Science, and Embase was carried out until August 2024. **Supplementary Table 1** provides a detailed search technique.

#### Inclusion and exclusion criteria, and data collection

Studies fulfilled the following requirements to be considered for inclusion: (1) they required a comparative analysis of survival outcomes (OS/PFS) among patients who experienced irAEs and those who did not. (2) they had to be observational studies or randomized controlled trials; and (3) the participants had to be adult cancer patients who had undergone at least one ICI treatment; and (4) The study needs to correct for time-shift bias. (1) Reviews, case reports, meta-analyses, and non-clinical research; (2) patients having surgery or other invasive procedures; and (3) studies without relevant clinical outcome data were among the exclusion criteria. Fundamental data, including author, country, sample size, year of publication, age, tumor type, type of ICIs, irAE categorization criteria, and survival outcome measures, were independently collected from the included papers by two researchers. Every piece of retrieved data was methodically documented using standardized forms, cross-checked, and any differences were settled by consensus. The primary results were 95% CIs and HR for OS or PFS, which were ideally computed using Kaplan-Meier survival curves or extracted from the articles.

#### Quality assessment and statistical analysis

To evaluate the caliber of the qualifying research, two authors employed the Newcastle-Ottawa Scale (NOS). This assessment was completed independently by each researcher, and disagreements were discussed and settled. Statistical procedures utilized R software (v.4.3.2). Study heterogeneity was quantified via I² statistics and Cochran’s Q testing. Random-effects models were implemented for substantial heterogeneity (I²>50%), otherwise fixed-effects approaches were applied.

## Results

### Cohort study

#### Basic patient characteristics

This retrospective cohort comprised 870 ICI-treated patients from two medical centers (2019-2024). Participants were stratified by irAE development status during treatment: non-irAEs group (n=588) and irAE groups (n=282). This cohort (**Table 1**) comprised 20.92% females (n=182) and 79.08% males (n=688), with identical median ages of 62 years (P>0.05). Lung cancer (395 cases, 45.40%) and gastrointestinal tumors (369 cases, 42.41%) were the most common tumor types. 94.8% of patients had an ECOG functional score of 0 and 1, according to baseline characteristics (13.22% scored 0, 81.95% scored 1), and there was a low prevalence of comorbidities (coronary heart disease 8.85%, diabetes mellitus 15.17%, hypertension 34.25%). In terms of treatment regimens, 88.28% (n=768) of patients received immunocombination chemotherapy, predominantly utilizing platinum-based regimens; 80.23% (n=698) were administered first-line immunotherapy, primarily with sindilizumab and karelizumab. Comprehensive results for the various subgroups are presented in **Table 1**.

**Table 1:** The clinical features of the cohort’s patients.

Variables	Total	Non-irAEs	irAEs	Statistic	P
	(n = 870)	(n = 588)	(n = 282)	(t/χ²)	
Age(Mean ± SD)	62.68 ± 9.36	62.28 ± 9.31	63.52 ± 9.40	-1.84	0.067
Gender, n(%)				0.29	0.592
Female	182 (20.92)	120 (20.41)	62 (21.99)		
Male	688 (79.08)	468 (79.59)	220 (78.01)		
Cancer, n(%)				0.35	0.840
Gastrointestinal cancer	369 (42.41)	252 (42.86)	117 (41.49)		
Lung cancer	395 (45.40)	263 (44.73)	132 (46.81)		
Other cancer	106 (12.18)	73 (12.41)	33 (11.70)		
Coronary heart disease, n(%)				2.37	0.120
No	793 (91.15)	542 (92.18)	251 (89.01)		
Yes	77 (8.85)	46 (7.82)	31 (10.99)		
Diabetes mellitus, n(%)				0.93	0.334
No	738 (84.83)	494 (84.01)	244 (86.52)		
Yes	132 (15.17)	94 (15.99)	38 (13.48)		
Hypertension, n(%)				0.96	0.330
No	572 (65.75)	393 (66.84)	179 (63.48)		
Yes	298 (34.25)	195 (33.16)	103 (36.52)		
Smoking, n(%)				0.00	0.957
Never	403 (46.32)	272 (46.26)	131 (46.45)		
Current or former	467 (53.68)	316 (53.74)	151 (53.55)		
Drinking, n(%)				0.28	0.600
Never	477 (54.83)	326 (55.44)	151 (53.55)		
Current or former	393 (45.17)	262 (44.56)	131 (46.45)		
ECOG-PS, n(%)				-	0.139
0	115 (13.22)	71 (12.07)	44 (15.60)		
1	713 (81.95)	493 (83.84)	220 (78.01)		
2	40 (4.60)	23 (3.91)	17 (6.03)		
3	2 (0.23)	1 (0.17)	1 (0.35)		
Metastatic site, n(%)				0.03	0.860
≤2	490 (56.32)	330 (56.12)	160 (56.74)		
>2	380 (43.68)	258 (43.88)	122 (43.26)		
Treatment line, n(%)				2.75	0.253
First line	698 (80.23)	466 (79.25)	232 (82.27)		
Second line	135 (15.52)	99 (16.84)	36 (12.77)		
Third line and above	37 (4.25)	23 (3.91)	14 (4.96)		
Chemotherapy, n(%)				1.79	0.181
No	102 (11.72)	63 (10.71)	39 (13.83)		
Yes	768 (88.28)	525 (89.29)	243 (86.17)		
irae Grading, n(%)				870	<0.001
No irAEs	588 (67.59)	588 (100.00)	0 (0.00)		
Grade 1-2	235 (27.01)	0 (0.00)	235 (83.33)		
Grade >2	47 (5.40)	0 (0.00)	47 (16.67)		
MLR, n(%)				0.00	0.998
≤0.47	654 (75.17)	442 (75.17)	212 (75.18)		
>0.47	216 (24.83)	146 (24.83)	70 (24.82)		
NLR, n(%)				1.47	0.225
≤3.45	527 (60.57)	348 (59.18)	179 (63.48)		
>3.45	343 (39.43)	240 (40.82)	240 (40.82)		
PLR, n(%)				0.22	0.637
≤186.98	496 (57.01)	332 (56.46)	164 (58.16)		
>186.98	374 (42.99)	256 (43.54)	118 (41.84)		
dNLR, n(%)				2.05	0.152
≤2.49	579 (66.55)	382 (64.97)	197 (69.86)		
>2.49	291 (33.45)	206 (35.03)	85 (30.14)		
SII, n(%)				0.01	0.920
≤888.32	544 (62.53)	367 (62.41)	177 (62.77)		
>888.32	326 (37.47)	221 (37.59)	105 (37.23)		
SIRI, n(%)				0.11	0.746
≤2.08	611 (70.23)	415 (70.58)	196 (69.50)		
>2.08	259 (29.77)	173 (29.42)	86 (30.50)		

SD, standard deviation; t, Student’s t-test; χ², Pearson’s chi-square test; - = Fisher’s exact test.

#### Immunotherapy-related toxicities

The study documented a total of 329 irAEs affecting 282 patients. The distribution of toxicities by system was as follows: endocrine toxicity was the most prevalent (83 cases, 25.2%), primarily hypothyroidism (71 cases, 21.6%); skin toxicity followed (78 cases, 23.7%), with skin rash accounting for 41 cases (12.5%); hepatotoxicity (58 cases, 17.6%) was mainly characterized by elevated aminotransferases (32 cases, 9.7%); and pulmonary toxicity (54 cases, 16.7%) was predominantly pneumonia (53 cases, 16.1%). Cardiovascular toxicity (22 cases, 6.7%) was primarily associated with elevated cardiac enzymes (12 cases, 3.6%). The majority of irAEs (83.9%, 276/329) were classified as grade 1-2, with only 16.1% (53/329) reaching grade 3–4 severity. Detailed results are provided in **Supplementary Table 2**.

#### irAEs’ effect on immunotherapy

**Figure 1:**
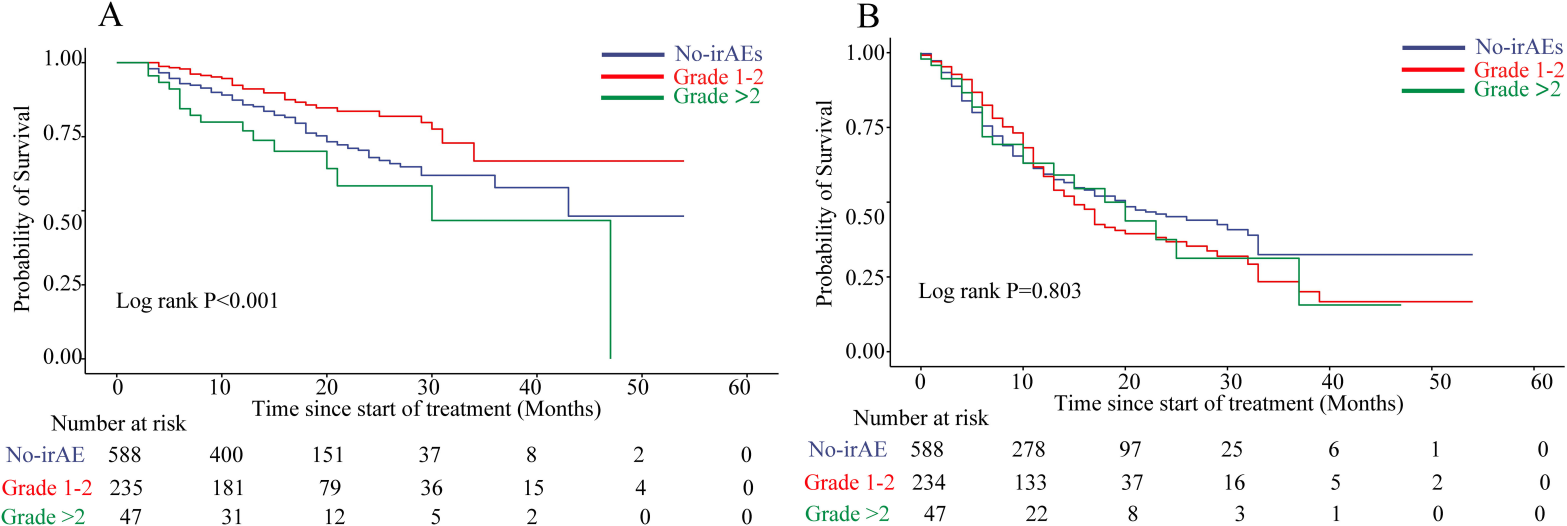
Association of irAE severity with improved survival outcomes. **(A)** Patients with higher-grade irAEs showed significantly improved OS. **(B)** No significant difference in PFS was observed across irAE severity groups. Statistical significance (p-values) was assessed via two-sided log-rank testing. CI, confidence interval; OS, overall survival; PFS, progression-free survival.

This investigation employed a time-dependent Cox proportional hazards regression model, incorporating irAEs as time-dependent covariates to evaluate their evolving influence on survival outcomes. The results indicated that patients who experienced irAEs demonstrated a statistically significant improvement in OS (HR = 0.974, 95% CI: 0.952–0.996, P = 0.022; see **Table 2**). Subsequent stratified analyses based on irAE severity revealed marked differences in OS across groups with varying irAE grades (log-rank P < 0.001; **Figure 1A**). Multivariate analysis identified high-grade (>2) irAEs as independent adverse prognostic factors for OS (HR = 1.93, 95% CI: 1.14–3.29, P = 0.015), whereas low-grade (1-2) irAEs were significantly correlated with enhanced OS (HR = 0.51, 95% CI: 0.34–0.76, P < 0.001; **Supplementary Table 3**). Importantly, no statistically significant differences were observed in PFS in relation to the presence of irAEs (HR = 1.016, 95% CI: 0.997–1.035, P = 0.108; **Table 2**) or when stratified by severity (log-rank P = 0.803, **Figure 1B**; **Supplementary Table 3**, P = 0.607).

**Table 2:** Association between immune-related adverse events and survival outcomes using time-dependent Cox regression analysis.

	Univariate analysis		Multivariate analysis^ab^	
	HR(95%CI)	p-value	HR(95%CI)	p-value
OS				
irAEs^a^	0.978(0.956,1.000)	0.510	0.974(0.952,0.996)	0.022
Endocrine toxicity^a^	0.942(0.910,0.974)	<0.001	0.938(0.905,0.971)	<0.001
Skin toxicity^a^	0.837(0.771,0.910)	<0.001	0.763(0.692,0.842)	<0.001
Hepatotoxicity^a^	1.324(1.032,1.698)	0.027	1.602(1.044,2.458)	0.031
Cardiotoxicity^a^	1.122(1.008,1.249)	0.035	1.181(1.030,1.355)	0.017
Pulmonary toxicity^a^	1.027(0.947,1.114)	0.517	1.089(0.980,1.211)	0.114
PFS				
irAEs^a^	1.019(1.000,1.038)	0.054	1.016(0.997,1.035)	0.108
Endocrine toxicity^a^	1.018(0.985,1.051)	0.298	1.025(0.990,1.061)	0.170
Skin toxicity^a^	0.944(0.868,1.026)	0.176	0.942(0.859,1.033)	0.203
Hepatotoxicity^a^	1.041(1.005,1.079)	0.024	1.044(1.005,1.086)	0.027
Cardiotoxicity^a^	1.109(1.022,1.204)	0.014	1.111(1.009,1.223)	0.032
Pulmonary toxicity^a^	1.015(0.983,1.048)	0.359	1.018(0.984,1.054)	0.307

HR, Hazard Ratio; CI, Confidence Interval; OS, Overall Survival; PFS, Progression-Free Survival.

a Time-dependent covariate.

b Separate multivariable Cox proportional hazards regression models were used, each incorporating a specific irAE category as a time-dependent covariate and adjusted for covariates including age, sex, tumor type, comorbidities (coronary heart disease, diabetes, hypertension), lifestyle factors (smoking, alcohol consumption), ECOG performance status, metastatic site, prior lines of therapy, and chemotherapy exposure.

#### Effects of various irAE types on survivability

In evaluating the influence of various organ-specific irAEs on OS and PFS among patients, PSM was initially employed to equilibrate baseline characteristics across groups. Post-matching, the distribution of samples within toxicity subgroups was rendered comparable (**Supplementary Figures 1A–E**), with a marked improvement in covariate balance evidenced by a general reduction in standardized mean differences (SMD) (**Supplementary Tables 4**-**8**). Subsequent time-dependent Cox proportional hazards modeling revealed that certain irAEs were significantly correlated with survival outcomes.

Survival analyses examining various categories of irAEs revealed considerable heterogeneity in their relationships with patient survival outcomes. Specifically, multivariate Cox regression analysis for OS demonstrated that endocrine toxicity (HR = 0.938, 95% CI: 0.905–0.971, P < 0.001) and skin toxicity (HR = 0.763, 95% CI: 0.692–0.842, P < 0.001) functioned as independent protective factors. In contrast, hepatotoxicity (HR = 1.602, 95% CI: 1.044–2.458, P = 0.031) and cardiotoxicity (HR = 1.181, 95% CI: 1.030–1.355, P = 0.017) were identified as independent risk factors for OS, whereas pulmonary toxicity did not exhibit a statistically significant association (P = 0.114). Regarding PFS, hepatotoxicity (HR = 1.044, 95% CI: 1.005–1.086, P = 0.027) and cardiotoxicity (HR = 1.111, 95% CI: 1.009–1.223, P = 0.032) were also independently associated with poorer prognosis. Conversely, endocrine, skin, and pulmonary toxicities showed no significant correlation with PFS. Comprehensive results are presented in **Table 2**.

#### irAEs’ effect on survival in various cancers

Due to their predominance in the cohort, this research specifically examined patients with gastrointestinal cancers and NSCLC. Analysis disclosed notable heterogeneity in irAE occurrence patterns among tumor types (**Supplementary Figure 2**). Among the 395 NSCLC patients, 132 (33.42%) experienced irAEs, with skin toxicity (38 cases, 28.79%) and endocrine toxicity (35 cases, 26.52%) being the most prevalent; in contrast, among the 369 gastrointestinal cancer patients, 117 (31.71%) experienced irAEs, with hepatotoxicity (36 cases, 30.77%) and pulmonary toxicity (25 cases, 21.37%) being the most common.

To mitigate the influence of potential confounding variables, propensity score matching was conducted independently within the NSCLC and gastrointestinal cancer cohorts, thereby achieving balanced baseline characteristics between patients experiencing irAEs and those without such events (refer to **Supplementary Figure 3**, **Supplementary Tables 9**, **10**). Subsequent survival analyses were performed on these matched cohorts. A time-dependent Cox proportional hazards model was employed to assess the relationship between irAE occurrence and survival outcomes, appropriately accounting for the temporal variability in irAE onset.

In two subgroup analyses involving patients with lung cancer and gastrointestinal cancer, multivariable Cox regression models revealed notable tumor-type heterogeneity in the prognostic significance of organ-specific irAEs. Among lung cancer patients, the presence of any irAEs was not significantly correlated with OS or PFS (OS: HR = 0.982, 95% CI: 0.944–1.021, p = 0.359; PFS: HR = 1.009, 95% CI: 0.982–1.038, p = 0.513). Furthermore, none of the organ-specific irAEs— including Pulmonary toxicity, hepatic toxicity, endocrine toxicity, dermal toxicity, and cardiac toxicity — demonstrated independent associations with OS or PFS in this cohort. Conversely, in patients with gastrointestinal cancer, the occurrence of any irAEs emerged as an independent protective factor associated with improved OS (HR = 0.946, 95% CI: 0.907–0.986, p = 0.009); however, this protective effect was not observed for PFS (HR = 1.028, 95% CI: 0.995–1.061, p = 0.096). Additionally, specific organ-related toxicities did not exhibit independent prognostic relevance within this subgroup. The results are shown in **Supplementary Tables 11**, **12**.

#### Impact of inflammatory markers on survival and adverse effects

To assess the prognostic utility of post-treatment inflammatory markers in predicting patient survival outcomes, we employed patient survival status as the endpoint and generated ROC curves for NLR, MLR, PLR, dNLR, SII, and SIRI) (see **Supplementary Table 13**). The analysis revealed that MLR exhibited the highest predictive accuracy, with an area under the curve (AUC) of 0.702 (95% CI: 0.653–0.751) and an optimal cutoff value of 0.47, yielding a sensitivity of 51.3% and specificity of 80.7%. Other markers, including NLR (AUC = 0.612), PLR (AUC = 0.604), and dNLR (AUC = 0.601), demonstrated moderate discriminatory capacity, whereas SII and SIRI showed limited predictive performance, with AUCs of 0.506 and 0.526, respectively. Based on the cutoff values derived from the ROC analyses (NLR = 3.45, MLR = 0.47, PLR = 186.98, dNLR = 2.49, SII = 888.32, SIRI = 2.08), patients were stratified into high and low groups for each inflammatory marker to further investigate their associations with survival prognosis.

**Figure 2:**
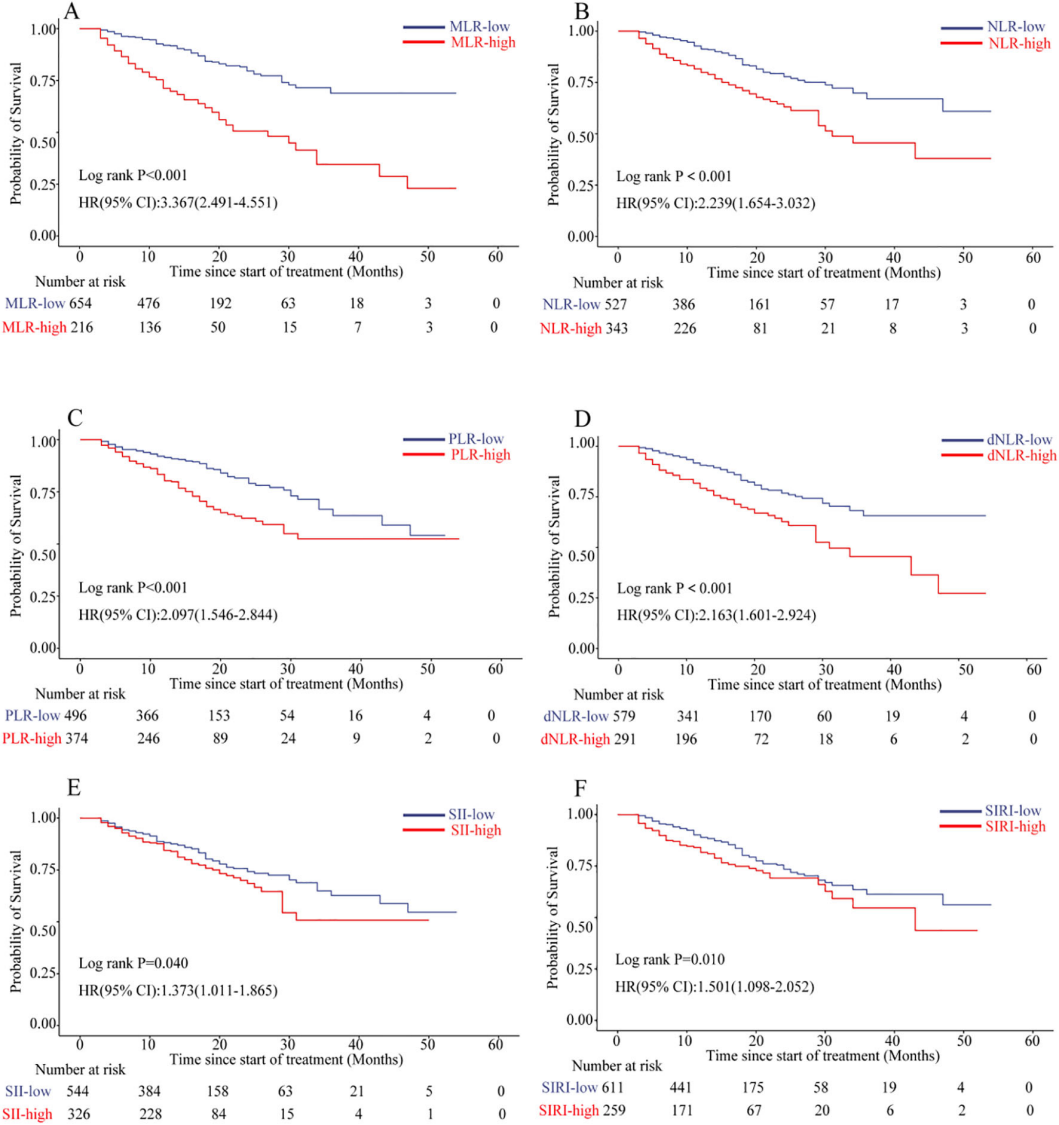
Kaplan-Meier analyses of overall survival (OS) stratified according to inflammatory marker thresholds: **(A)** MLR, **(B)** NLR, **(C)** PLR, **(D)** dNLR, **(E)** SII, **(F)** SIRI. Statistical significance was determined by two-sided log-rank testing. CI, confidence interval; MLR, monocyte-lymphocyte ratio; NLR, neutrophil-lymphocyte ratio; PLR, platelet-lymphocyte ratio; dNLR, derived neutrophil-lymphocyte ratio; SII, systemic immune-inflammation index; SIRI, systemic inflammation response index.

Survival analysis using Kaplan-Meier curves grouped by inflammatory markers and confirmed by the log-rank test demonstrated that patients with an MLR greater than 0.47 had significantly shorter OS and PFS (both P < 0.001; see **Figure 2**; **Supplementary Figure 4**). In univariate analysis, higher levels of all six inflammatory markers were significantly linked to worse OS. Among them, MLR had the highest hazard ratio (HR = 3.37, 95% CI: 2.49-4.55, P < 0.001), followed by NLR (HR = 2.24, 95% CI: 1.65-3.03, P < 0.001), PLR (HR = 2.10, 95% CI: 1.55-2.84, P < 0.001), and dNLR (HR = 2.16, 95% CI: 1.60-2.92, P < 0.001). SII (HR = 1.37, 95% CI: 1.01-1.86, P = 0.040) and SIRI (HR = 1.50, 95% CI: 1.10-2.05, P = 0.011) also showed statistically significant associations (**Figure 2**). After adjusting for confounding variables in a multivariable Cox proportional hazards model, only MLR and PLR remained as independent prognostic factors for OS (**Supplementary Table 14**). Specifically, an MLR above 0.47 was the strongest independent risk factor for OS (HR = 3.05, 95% CI: 2.11-4.41, P < 0.001) and also retained independent significance in the multivariable model for PFS (HR = 1.59, 95% CI: 1.22-2.07, P < 0.001). A PLR above 186.98 was also associated with significantly reduced OS (HR = 1.75, 95% CI: 1.20-2.56, P = 0.004), although its link to PFS was not significant in the multivariable analysis. The other inflammatory markers—NLR, dNLR, SII, and SIRI—did not demonstrate independent prognostic value in the multivariable analysis (all P > 0.05) (**Supplementary Table 14**).

Additionally, exploratory analyses indicated that baseline inflammatory marker levels were not significantly associated with the incidence of irAEs (all P > 0.05; see **Supplementary Table 15**).

### Meta-analysis results

In accordance with the predefined retrieval strategy, an initial total of 13,057 records were obtained from multiple databases. Subsequent to the removal of duplicate entries and screening of titles and abstracts, 278 articles were identified for full-text review. After a thorough eligibility assessment 38 studies met the inclusion criteria and were incorporated into this meta-analysis, encompassing a combined sample of 55,966 patients. Comprehensive details regarding the characteristics of all included studies are provided in **Supplementary Figure 5** and **Supplementary Table 16**.

The final analysis encompassed 38 studies, the majority of which were retrospective in nature (n=35), alongside four prospective investigations. Most studies evaluated inhibitors targeting PD-1 and/or PD-L1. Geographically, the Asia-Pacific region and Europe are the most prominent, with 13 studies each, followed by North America (10 entries) and other regions (2 entries). The median sample size across studies was 223 patients, with an interquartile range of 130 to 621. Comprehensive details of all included studies are available in **Supplementary Table 16**.

**Figure 3:**
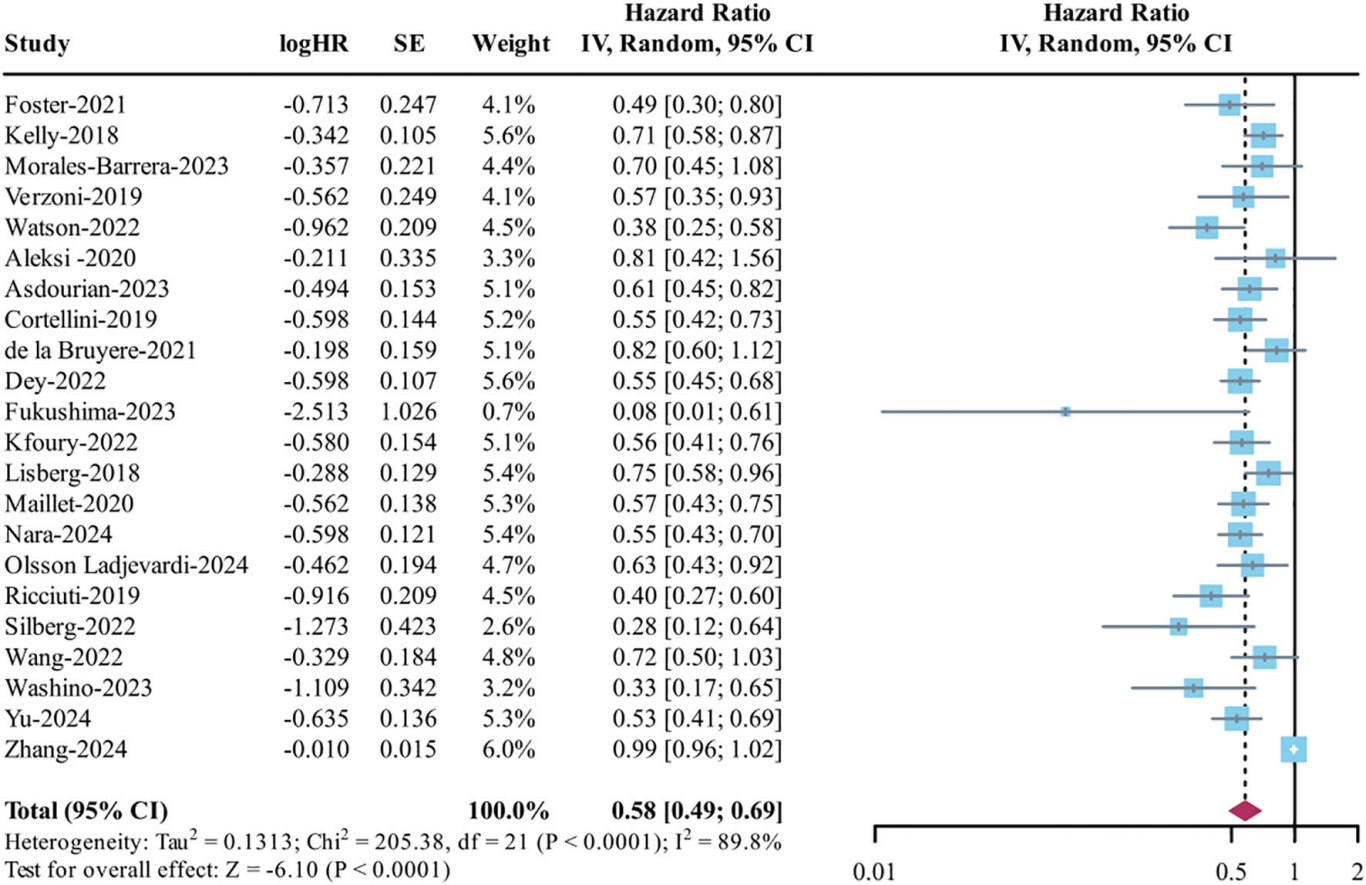
Analysis of overall survival (OS) between irAEs and ICI efficacy.

**Figure 4:**
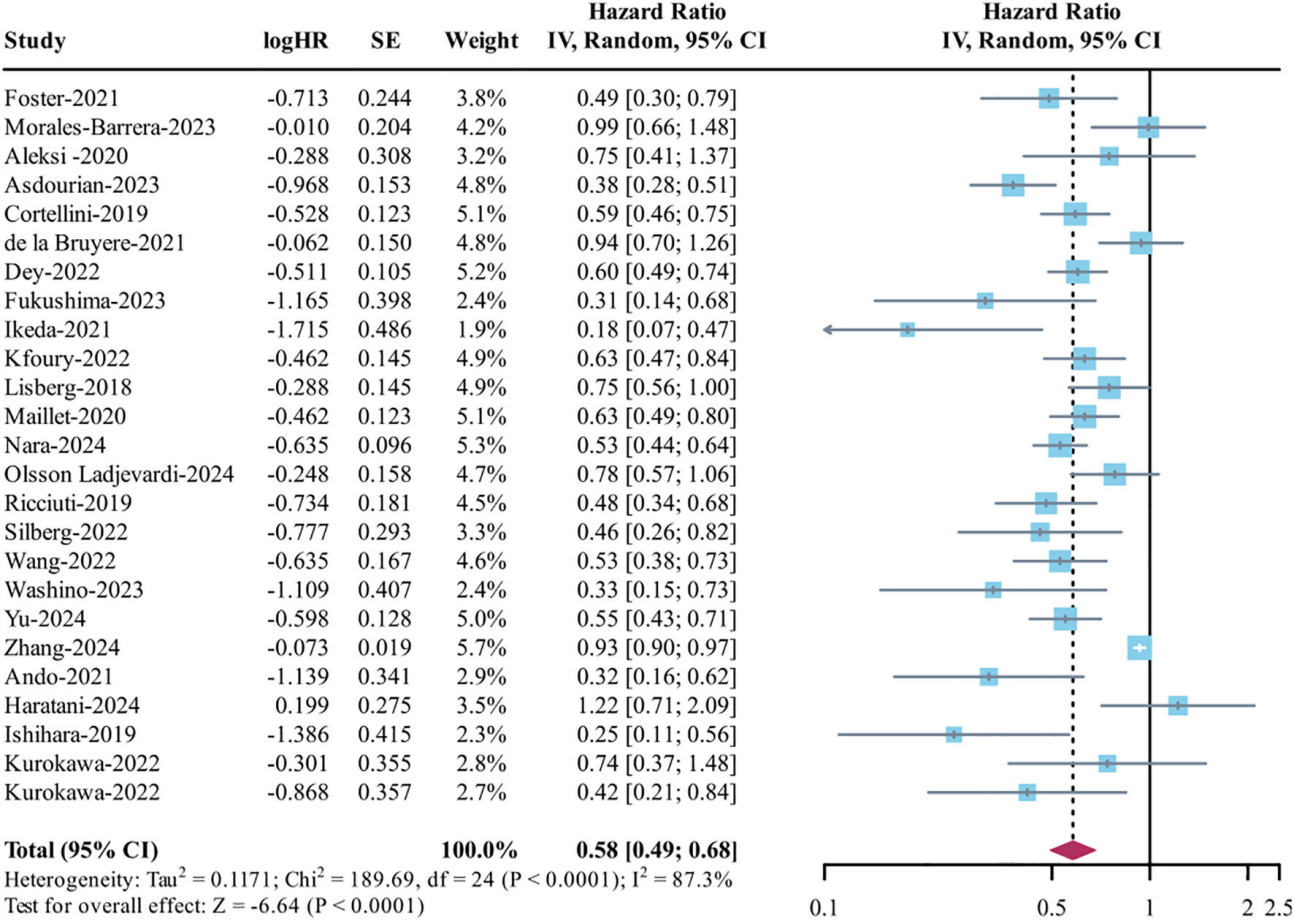
Analysis of progression-free survival (PFS) between irAEs and ICI efficacy.

This study found considerable variability in the odds ratios (I² = 89.8%), so a random-effects model was applied for the combined analysis. The findings revealed that patients who experienced irAEs had significantly better OS compared to those who did not (HR = 0.58, 95% CI: 0.49–0.69, p < 0.0001) (see **Figure 3**). Further subgroup analysis showed that improvements in OS were significantly linked to both high-grade (>2: HR = 0.70, 95% CI: 0.62–0.83, I² = 46.5%) and low-grade (≤2: HR = 0.54, 95% CI: 0.46–0.64, I² = 33.3%) irAEs (refer to **Supplementary Figure 6**). When examining different types of irAEs, any irAE, as well as endocrine, gastrointestinal, and skin toxicities, were significantly associated with better OS (all p < 0.001), while pneumonitis (p = 0.078) and hepatobiliary toxicity (p = 0.79) did not show a significant relationship (see **Supplementary Figure 7**). There was also notable heterogeneity in PFS analysis (I² = 87.3%), leading to the use of a random-effects model for pooling data, which demonstrated improved PFS in the irAE group (HR = 0.58, 95% CI: 0.49–0.68, p < 0.0001) (**Figure 4**). In the severity subgroup, patients with irAEs of grade ≤2 showed significantly better PFS (HR = 0.51, 95% CI: 0.30–0.87, I² =85%, p = 0.013) (**Supplementary Figure 8**). Additionally, any irAE, along with endocrine, gastrointestinal, and skin toxicities, were significantly linked to PFS benefits (all p < 0.001), whereas pneumonitis (p = 0.51) and hepatobiliary toxicity (p = 0.39) had no significant impact (**Supplementary Figure 9**).

## Discussion

This investigation combines real-world cohort data with meta-analytic approaches to systematically elucidate the complex and multifaceted relationship between irAEs and patient survival outcomes. The findings demonstrate that the prognostic impact of irAEs is markedly influenced by both their severity and the specific organs affected: high-grade irAEs (greater than Grade 2) serve as independent adverse prognostic indicators. Organ-specific analyses reveal that hepatotoxicity and cardiotoxicity are associated with a significantly elevated risk of mortality, whereas skin toxicity and endocrine toxicity appear to confer a protective effect. Further stratified analyses indicate variability in these associations across distinct cancer types, such as non-small cell lung cancer and gastrointestinal malignancies. Moreover, post-treatment inflammatory biomarkers—including the MLR, PLR, and NLR—emerged as significant independent prognostic factors. Collectively, these results highlight the imperative of nuanced stratification of irAEs alongside systemic inflammatory status in clinical settings to facilitate the development of personalized prognostic evaluation frameworks.

The observed positive correlation between adverse effects and therapeutic efficacy may illustrate the “double-edged sword” nature of the biological effects of pharmacological agents. Utilizing a time-dependent Cox proportional hazards model, this study demonstrated that the incidence of irAEs is significantly associated with enhanced OS in patients. This finding aligns with the majority of existing literature, thereby reinforcing the hypothesis that irAEs may function as potential biomarkers indicative of ICI therapeutic efficacy (19–21). Regarding organ-specific irAEs, the findings of this study contribute significantly to the ongoing discourse within the existing body of evidence. Prior research has indicated that organ-specific irAEs exhibit heterogeneous effects on survival outcomes; specifically, endocrine and musculoskeletal irAEs are associated with survival benefits, while gastrointestinal, pneumonitis, neurologic, hepatic, renal, and other organ-specific irAEs negatively impact survival, suggesting that different organ-specific irAEs correlate with varying prognostic outcomes (22). The subgroup analysis within this study revealed that the incidence of endocrine toxicity was 25.2%, with primary clinical manifestations including hypothyroidism (21.6%), hyperthyroidism (2.4%), and hyperglycemia (0.9%), a distribution consistent with previous literature (23). This investigation, utilizing a time-dependent multivariable Cox regression analysis, substantiated that hepatotoxicity constitutes an independent prognostic factor for patient survival, thereby reinforcing evidence previously reported in the literature (24). Conversely, skin irAEs were linked to a significant extension of both OS and PFS, corroborating findings from earlier studies (25). The multivariate analysis conducted in this study did not reveal a statistically significant negative impact of pneumonia irAEs on patient survival. This outcome contrasts with existing literature, which presents conflicting views on this matter. One potential explanation for this discrepancy is that pneumonia irAEs, while potentially severe or fatal, may have adverse effects that are partially counterbalanced by the therapeutic advantages conferred by ICIs (26). This could elucidate the absence of significant survival detriments observed in the current study. Additionally, the inclusion of patients with advanced NSCLC, who frequently present with pre-existing lung conditions, may have further diminished the efficacy of ICIs. Moreover, the literature exhibits a notable divergence regarding the relationship between gastrointestinal irAEs (such as colitis or diarrhea) and survival benefits. Some studies report no significant survival advantage, while others indicate that these irAEs may be predictive of improved OS and PFS (27). This inconsistency may stem from variations in the baseline intestinal flora among patients (28, 29). However, the present study was unable to adequately evaluate this association due to the limited number of gastrointestinal irAEs documented. Furthermore, irAEs are hypothesized to signify a reactivation of the immune system, prompting an investigation into whether patients experiencing severe irAEs exhibit improved outcomes compared to those with mild irAEs. Consistent with our hypothesis, although the treatment did not significantly improve PFS, patients who developed grade >2 irAEs had shorter OS than those without, in agreement with prior reports (30, 31). This finding is particularly noteworthy as it contrasts with the results of several studies, which indicated that patients with severe irAEs (grades 3-5) had significantly enhanced PFS and OS compared to those with mild irAEs (30). This discrepancy suggests that the relationship between the severity of toxicity and survival benefit may be influenced by various factors, including the intensity of the therapeutic intervention and the underlying disease status (32).

Beyond adverse drug reactions, systemic inflammatory status plays a crucial role in modulating therapeutic responses in cancer treatment. Blood-based inflammatory indices (NLR, PLR, MLR) serve as established prognostic biomarkers for immune checkpoint inhibitors across malignancies including colorectal, NSCLC, and renal carcinoma (33–35). In advanced malignancies receiving anti-PD-1 treatment, inflammatory index dynamics are poorly defined. Our analysis revealed that NLR, PLR, and MLR were significantly correlated with OS. These findings align with observations reported in other oncological patient populations. Research by Ziting Ou et al. (35) further demonstrated that NLR, PLR, and MLR could assess short-term immunotherapy responses in anti-PD-1-treated gastric cancer cases, while Mingyu Wan et al. (36) established NLR as a robust predictor of PFS/OS in advanced GC patients receiving chemoimmunotherapy. Collectively, these findings underscore the cancer-specific dependence of the predictive value of inflammatory markers, necessitating careful individual consideration when selecting optimal biomarkers for personalized treatment. In the current investigation of the relationship between inflammatory markers and irAEs, the findings revealed no significant correlation, which diverges from the conclusions of prior research. Earlier studies have indicated that the NLR is markedly elevated in patients with NSCLC experiencing irAEs, and it has been identified as a potential biomarker due to its accessibility and cost-effectiveness (37). A similar trend has been observed in patients with cardiac adverse events, as evidenced by elevated NLR levels in individuals experiencing Major Adverse Cardiovascular Events, as reported by Moey et al (38). Additionally, elevated NLR and C-reactive protein levels have been associated with immune-related pneumonial (39). However, the present study did not replicate these associations, which may be attributed to the heterogeneity introduced by the multicancer cohort encompassing various subtypes of irAEs.

In interpreting the aforementioned findings, it is imperative to thoroughly consider the treatment context of the studied cohort, wherein the vast majority of patients (88.3%) received immune checkpoint inhibitors in combination with chemotherapy. This prevalent therapeutic approach introduces additional complexity to the elucidation of the relationship between irAEs and treatment efficacy. Specifically, multiple synergistic mechanisms exist between chemotherapy and immunotherapy: chemotherapeutic agents not only amplify and deepen T-cell responses via the induction of immunogenic cell death but also potentiate antitumor immunity by modulating pathways associated with the PD-(L)1 axis, such as the inhibition of STAT signaling and the augmentation of the cytotoxic T cell to regulatory T cell rati (40). Furthermore, evidence indicates that chemotherapeutic drugs such as pemetrexed can elicit systemic immune activation by promoting T-cell stimulation and increasing the susceptibility of cancer cells to immune-mediated cytotoxicity (41). These mechanisms may partially explain the observation that, within the context of combination therapy, certain mild to moderate irAEs (e.g., dermatologic and endocrine toxicities) remain associated with significant survival benefits. Clinical data support the potential advantages of combination regimens. For instance, a retrospective analysis of patients with PD-L1 expression ≥50% revealed no statistically significant difference in overall survival between the pembrolizumab monotherapy and combination therapy groups (13.3 months versus 20.4 months, P = 0.18). However, the combination therapy cohort showed a trend toward improved tumor response rates (42). Nonetheless, the use of combination therapy complicates the attribution of adverse events, as chemotherapy-induced toxicities (such as elevated hepatic enzymes and diarrhea) may overlap with clinical manifestations of irAEs, potentially resulting in underestimation or misclassification of irAE incidence. Consequently, the conclusions drawn from this study should be interpreted within the context of combination therapy as the prevailing clinical paradigm. Future prospective investigations are warranted to differentiate among treatment modalities, thereby elucidating the independent prognostic impact of irAEs in the settings of immune monotherapy versus combination therapy.

Underlying these clinical observations is a potential dual regulatory network involving systemic inflammation and immunotoxicity. Although the precise mechanisms of irAEs remain incompletely understood, there is a prevailing consensus that they represent a bystander effect of activated T cells, aligning with the action mechanism of ICIs: the antigen-specific T cells that are activated inadvertently result in the targeting of host tissues, accompanied by B-cell-mediated autoantibody production and a surge of inflammatory cytokines, ultimately resulting in organ-specific inflammation. Recent advances have further clarified the critical regulatory role of myeloid immune cells in irAEs. Notably, a post-mortem study of a patients with melanoma experiencing fulminant myocarditis following treatment with nivolumab and ipilimumab revealed significant T-cell infiltration of the heart muscle and pronounced M1 macrophage polarization (43). This finding directly demonstrates that PD-1 inhibitors can mediate cardiotoxicity by modulating macrophage polarization states (43). Additionally, the association between the NLR and ICI outcomes may stem from the pivotal role of neutrophils within the tumor microenvironment (44, 45). Neutrophils extensively infiltrate the tumor microenvironment (TME), and their excessive proliferation leads to the secretion of various pro-tumorigenic substances, promoting TME formation and influencing immune responses (46). Furthermore, neutrophils are strongly implicated in tumorigenesis, progression, and early metastasis (47).

This study is not without limitations. Its retrospective design potentially introduces selection and information biases. Although propensity score matching and time-dependent multivariable Cox regression models were utilized to mitigate the impact of known confounding factors, the possibility of residual confounding due to unmeasured variables cannot be entirely excluded. The study population is relatively homogeneous, and certain subgroup sample sizes are limited, which may compromise the stability and generalizability of the findings. Additionally, although the diagnosis of irAEs adhered to established guidelines, challenges persist in achieving consistent assessment of subjective symptoms. Moreover, the prevalent use of combination therapies further complicates the attribution of adverse events. The meta-analysis, encompassing 38 retrospective studies, may have reduced reliability due to heterogeneity in reporting and the lack of comparability in baseline characteristics across studies.

## Conclusion

In summary, evidence derived from clinical trials and meta-analyses indicates that the range of organs affected, the severity of irAEs, and systemic inflammatory markers significantly impact the efficacy of immunotherapy. Notably, skin toxicity and endocrine toxicity are generally associated with a favorable prognosis, whereas cardiovascular toxicities, hepatotoxicity and irAEs of grade ≥3 correlate with a poorer prognosis. It is important to highlight that, although inflammatory markers possess independent predictive value regarding efficacy, their influence is not contingent upon the occurrence of irAEs. Given the limitations inherent in the current study, there is a pressing need for large-scale prospective investigations and an exploration of the underlying mechanisms to further elucidate the regulatory pathways governing irAEs and inflammatory responses in relation to immunotherapy efficacy. The objective of this study is to establish a robust evidence-based framework for enhancing risk stratification management of irAEs, ultimately aiming to improve the clinical outcomes for cancer patients.

## Data Availability

The raw data supporting the conclusions of this article will be made available by the authors, without undue reservation.
