# Supplementary material for: Immune checkpoint inhibitor therapy in advanced cancer: clinical association of irAEs type, inflammatory markers and efficacy
Source: Front Immunol. 2025 Nov 26;16:1662333. doi: 10.3389/fimmu.2025.1662333 (PMC12689405; doi:10.3389/fimmu.2025.1662333)
Supplement: Supplementary file 1 [file Table1.docx]

**Supplementary Table 1** Literature screening search format details

| Literature Database | Literature Search Format | Number of Searches |
| --- | --- | --- |
| Pubmed |  | Deadline 2024-08-20 |
| 1 | ((((((((((((Immune Checkpoint Inhibitor[MeSH Terms])) OR (immune checkpoint blockers[Title/Abstract])) OR (ICI[Title/Abstract])) OR (PD 1 inhibitor[Title/Abstract])) OR (PD L1 inhibitor[Title/Abstract])) OR (nivolumab[Title/Abstract])) OR (Pembrolizumab[Title/Abstract])) OR (Sintilimab[Title/Abstract])) OR (Atezolizumab[Title/Abstract])) OR (Avelumab[Title/Abstract])) OR (Durvalumab[Title/Abstract])) OR (Immunotherapy[Title/Abstract]) OR (ctla-4 inhibitor[Title/Abstract]) OR (ipilimumab[Title/Abstract]) | 174862 |
| 2 | (((((((Drug-Related Side Effects and Adverse Reactions[MeSH Terms]) OR (Immune-related adverse events[Title/Abstract])) OR (Immune-related adverse reaction[Title/Abstract])) OR (irAEs[Title/Abstract])) OR (Drug Side Effect[Title/Abstract])) OR (Adverse Event[Title/Abstract])) OR (Adverse reaction[Title/Abstract])) OR (Drug Toxicity[Title/Abstract]) | 196263 |
| 3 | (((Efficacy[Title/Abstract]) OR (Outcome[Title/Abstract])) OR (Prognosis[Title/Abstract])) OR (survival[Title/Abstract]) | 3674081 |
| 4 | #1 and #2 and #3 | 4102 |
| 5 | [#4 Filters applied: Humans](https://pubmed.ncbi.nlm.nih.gov/?term=((((((((((((((Immune+Checkpoint+Inhibitor%5bMeSH+Terms%5d))+OR+(immune+checkpoint+blockers%5bTitle/Abstract%5d))+OR+(ICI%5bTitle/Abstract%5d))+OR+(PD+1+inhibitor%5bTitle/Abstract%5d))+OR+(PD+L1+inhibitor%5bTitle/Abstract%5d))+OR+(nivolumab%5bTitle/Abstract%5d))+OR+(Pembrolizumab%5bTitle/Abstract%5d))+OR+(Sintilimab%5bTitle/Abstract%5d))+OR+(Atezolizumab%5bTitle/Abstract%5d))+OR+(Avelumab%5bTitle/Abstract%5d))+OR+(Durvalumab%5bTitle/Abstract%5d))+OR+(Immunotherapy%5bTitle/Abstract%5d)+OR+(ctla-4+inhibitor%5bTitle/Abstract%5d)+OR+(ipilimumab%5bTitle/Abstract%5d))+AND+((((((((Drug-Related+Side+Effects+and+Adverse+Reactions%5bMeSH+Terms%5d)+OR+(Immune-related+adverse+events%5bTitle/Abstract%5d))+OR+(Immune-related+adverse+reaction%5bTitle/Abstract%5d))+OR+(irAEs%5bTitle/Abstract%5d))+OR+(Drug+Side+Effect%5bTitle/Abstract%5d))+OR+(Adverse+Event%5bTitle/Abstract%5d))+OR+(Adverse+reaction%5bTitle/Abstract%5d))+OR+(Drug+Toxicity%5bTitle/Abstract%5d)))+AND+((((Efficacy%5bTitle/Abstract%5d)+OR+(Outcome%5bTitle/Abstract%5d))+OR+(Prognosis%5bTitle/Abstract) | 3001 |
| WOS |  |  |
| 1 | (((((((TI=(Drug-Related Side Effects and Adverse Reactions)) OR TI=(Immune-related adverse events)) OR TI=(Immune-related adverse reaction)) OR TI=(irAEs)) OR TI=(Drug Side Effect)) OR TI=(Adverse Event)) OR TI=(Adverse reaction)) OR TI=(Drug Toxicity) and Preprint Citation Index (Exclude – Database) | 52806 |
| 2 | (((((((((((((TI=(Immune Checkpoint Inhibitor )) OR TI=(immune checkpoint blockers)) OR TI=(ICI)) OR TI=(PD 1 inhibitor)) OR TI=(PD L1inhibitor)) OR TI=(CTLA-4 inhibitor)) OR TI=(nivolumab)) OR TI=(Pembrolizumab)) OR TI=(Sintilimab)) OR TI=(Atezolizumab)) OR TI=(Avelumab)) OR TI=(Durvalumab)) OR TI=(ipilimumab)) OR TI=(Immunotherapy) and Preprint Citation Index (Exclude – Database) | 116184 |
| 3 | (((TI=(Efficacy)) OR TI=(Outcome)) OR TI=(Prognosis)) OR TI=(survival) and Preprint Citation Index (Exclude – Database) | 1482331 |
| 4 | #1 and #2 and #3 | 394 |
| Cochrane Library |  |  |
| 1 | MeSH descriptor: [Drug-Related Side Effects and Adverse Reactions] explode all trees | 5231 |
| 2 | (Immune-related adverse events OR Immune-related adverse reaction OR irAEs OR Drug Side Effect OR Adverse Event OR Adverse reaction OR Drug Toxicity):ti,ab,kw | 190820 |
| 3 | #1 or #2 |  |
| 4 | MeSH descriptor: [Immune Checkpoint Inhibitors] explode all trees | 193283 |
| 5 | (immune checkpoint blockers OR ICI OR PD 1 inhibitor OR PD L1 inhibitor OR CTLA-4 inhibitor OR nivolumab OR Pembrolizumab OR Sintilimab OR Atezolizumab OR Avelumab OR Durvalumab OR ipilimumab OR immunotherapy):ti,ab,kw | 25371 |
| 6 | #4 or #5 | 25408 |
| 7 | (Efficacy or Outcome or Prognosis or survival):ti,ab,kw | 1063822 |
| 8 | #3 and #6 and #7 | 6726 |
| Embase |  |  |
| 1 | adverse drug reaction'/exp | 684907 |
| 2 | drug reated side eftecs':ti,ab,kw OR 'adverse reactions':ti,ab,kw OR 'immune-related adverse events':ti,ab,kw OR 'imune-related adverse reacion':ti,ab,kw OR 'irAEs':ti,ab,kw OR 'drug side effect':ti,ab,kw OR 'adverse event':ti,ab,kw OR "adverse reaction' ti,ab,kw OR 'drug toxicity' :ti,ab,kw | 113827 |
| 3 | #1 or #2 | 768248 |
| 4 | immune checkpoint inhibitor'/exp | 34616 |
| 5 | immune ctheckpoint blocker':ti,ab,kw OR 'ICI':ti,ab,kw OR 'pd 1 inhibtor':ti,ab,kw OR 'pd l1 inhibitor':ti,ab,kw OR 'ctla-4 inhibitor':ti,ab,kw OR 'nivoumab':ti,ab,kw OR 'pembrolizumab':ti,ab,kw OR 'sinitimabtiab':ti,ab,kw OR 'atezolizumab':ti,ab,kw OR 'avelumab':ti,ab,kw OR 'durvalumab':ti,ab,kw OR 'ipilimumab':ti,ab,kw OR 'immunotherapy':ti,ab ,kw | 272422 |
| 6 | #4 or #5 | 283307 |
| 7 | efficacy':ti,ab,kw OR 'outcome':ti,ab,kw OR 'prognosis':ti,ab,kw OR 'survival':ti,ab,kw | 5342172 |
| 8 | #3 and #6 and #7 | 17670 |
| 9 | #8 AND 'human'/de | 17270 |
| 10 | #9 AND 'human'/de AND 'article'/it | 6252 |

**Supplementary Table 2** Spectrum and grading characteristics of adverse event safter PD-1/PD-L1 inhibitor therapy(n=318)

|  | Grade 1 | Grade 2 | Grade ≥3 |
| --- | --- | --- | --- |
| Endocrine toxicity | 9 | 73 | 1 |
| Hypothyroidism | 4 | 66 | 1 |
| Hyperthyroidism | 4 | 4 | 0 |
| Hyperglycemia | 1 | 2 | 0 |
| Hypopituitarism | 0 | 1 | 0 |
| Dermatologic toxicity | 17 | 48 | 13 |
| Skin rash | 6 | 26 | 9 |
| Capillary hyperplasia | 3 | 5 | 0 |
| Pruritus | 4 | 5 | 0 |
| Hemangioma | 1 | 3 | 0 |
| Dermatitis | 0 | 2 | 1 |
| Psoriasis | 0 | 3 | 0 |
| Mucosal ulceration | 2 | 1 | 0 |
| Other dermatologic toxicities | 1 | 3 | 3 |
| Hepatotoxicity | 14 | 36 | 8 |
| Elevated transaminases | 10 | 20 | 2 |
| Abnormal liver function | 3 | 12 | 4 |
| Hepatitis | 1 | 4 | 2 |
| Pulmonary toxicity | 3 | 40 | 11 |
| Pneumonia | 2 | 40 | 11 |
| Dry cough | 1 | 0 | 0 |
| Cardiovascular toxicity | 3 | 8 | 11 |
| Elevated cardiac enzymes | 3 | 4 | 5 |
| Myocarditis | 0 | 2 | 5 |
| Atrial premature beats | 0 | 2 | 0 |
| Hypertension | 0 | 0 | 1 |
| Systemic toxicity | 10 | 6 | 0 |
| Fever | 9 | 4 | 0 |
| Fatigue | 1 | 2 | 0 |
| Nephrotoxicity | 0 | 5 | 5 |
| Nephritis | 0 | 1 | 5 |
| Elevated creatinine | 0 | 4 | 0 |
| Hematologic toxicity | 0 | 3 | 1 |
| Myelosuppression | 0 | 1 | 0 |
| Coagulopathy | 0 | 1 | 1 |
| Leukopenia | 0 | 1 | 0 |
| Gastrointestinal toxicity | 0 | 0 | 3 |
| Enteritis | 0 | 0 | 1 |
| Pancreatitis | 0 | 0 | 1 |
| Diarrhea | 0 | 0 | 1 |
| Infusion reaction | 0 | 1 | 0 |

**Supplementary Table 3** Analysis of the association between different irAE grades and patient survival outcomes

|  | OS | | PFS | |
| --- | --- | --- | --- | --- |
|  | HR(95%CI) | p-value | HR(95%CI) | p-value |
| irAE grading ( vs. Non-irAE ) |  |  |  |  |
| Grand 1-2 | 0.51 (0.34 ~ 0.76) | <0.001 | 1.06 (0.85 ~ 1.32) | 0.607 |
| Grand ＞2 | 1.93 (1.14 ~ 3.29) | 0.015 | 1.25 (0.81 ~ 1.95) | 0.315 |
| Gender (vs. Female) | 0.82 (0.52 ~ 1.29) | 0.387 | 0.87 (0.65 ~ 1.17) | 0.355 |
| Age | 1.01 (0.99 ~ 1.02) | 0.536 | 0.99 (0.98 ~ 1.00) | 0.058 |
| Gastrointestinal cancer (vs. No) | 0.93 (0.57 ~ 1.53) | 0.785 | 0.68 (0.48 ~ 0.97) | 0.036 |
| Lung cancer (vs. No) | 0.58 (0.35 ~ 0.97) | 0.038 | 0.82 (0.58 ~ 1.16) | 0.265 |
| Coronaryheart disease (vs. No) | 0.82 (0.48 ~ 1.41) | 0.472 | 0.67 (0.45 ~ 0.99) | 0.048 |
| Diabete smellitus (vs. No) | 0.89 (0.63 ~ 1.24) | 0.481 | 0.94 (0.76 ~ 1.17) | 0.601 |
| Hypertension (vs. No) | 1.37 (0.91 ~ 2.05) | 0.127 | 1.41 (1.07 ~ 1.86) | 0.014 |
| Smoking (vs. No) | 0.93 (0.58 ~ 1.49) | 0.753 | 1.33 (1.00 ~ 1.79) | 0.052 |
| Drinking (vs. No) | 1.43 (0.92 ~ 2.23) | 0.109 | 1.33 (1.00 ~ 1.79) | 0.954 |
| ECOG_PS (vs. 0) |  |  |  |  |
| 1 | 0.63 (0.43 ~ 0.93) | 0.019 | 0.93 (0.70 ~ 1.24) | 0.62 |
| 2 | 1.01 (0.50 ~ 2.02) | 0.986 | 0.88 (0.51 ~ 1.53) | 0.656 |
| 3 | 6.34 (0.84 ~ 47.72) | 0.073 | 9.80 (2.34 ~ 40.93) | 0.002 |
| Metastatic site (＞2 vs. ≤2) | 1.67 (1.19 ~ 2.34) | 0.003 | 1.12 (0.91 ~ 1.38) | 0.271 |
| Treatment line (vs. First line) |  |  |  |  |
| Second line | 1.69 (1.16 ~ 2.47) | 0.007 | 1.26 (0.96 ~ 1.65) | 0.096 |
| Third line and above | 2.39 (1.31 ~ 4.39) | 0.005 | 2.62 (1.71 ~ 4.02) | <0.001 |
| Chemotherapy (vs. No) | 0.76 (0.48 ~ 1.21) | 0.242 | 1.48 (1.03 ~ 2.13) | 0.034 |
| HR: Hazard Ratio, CI: Confidence Interval, OS: Overall Survival, PFS:Progression-Free Survival | | | | |

| **A** | **B** |
| --- | --- |
| **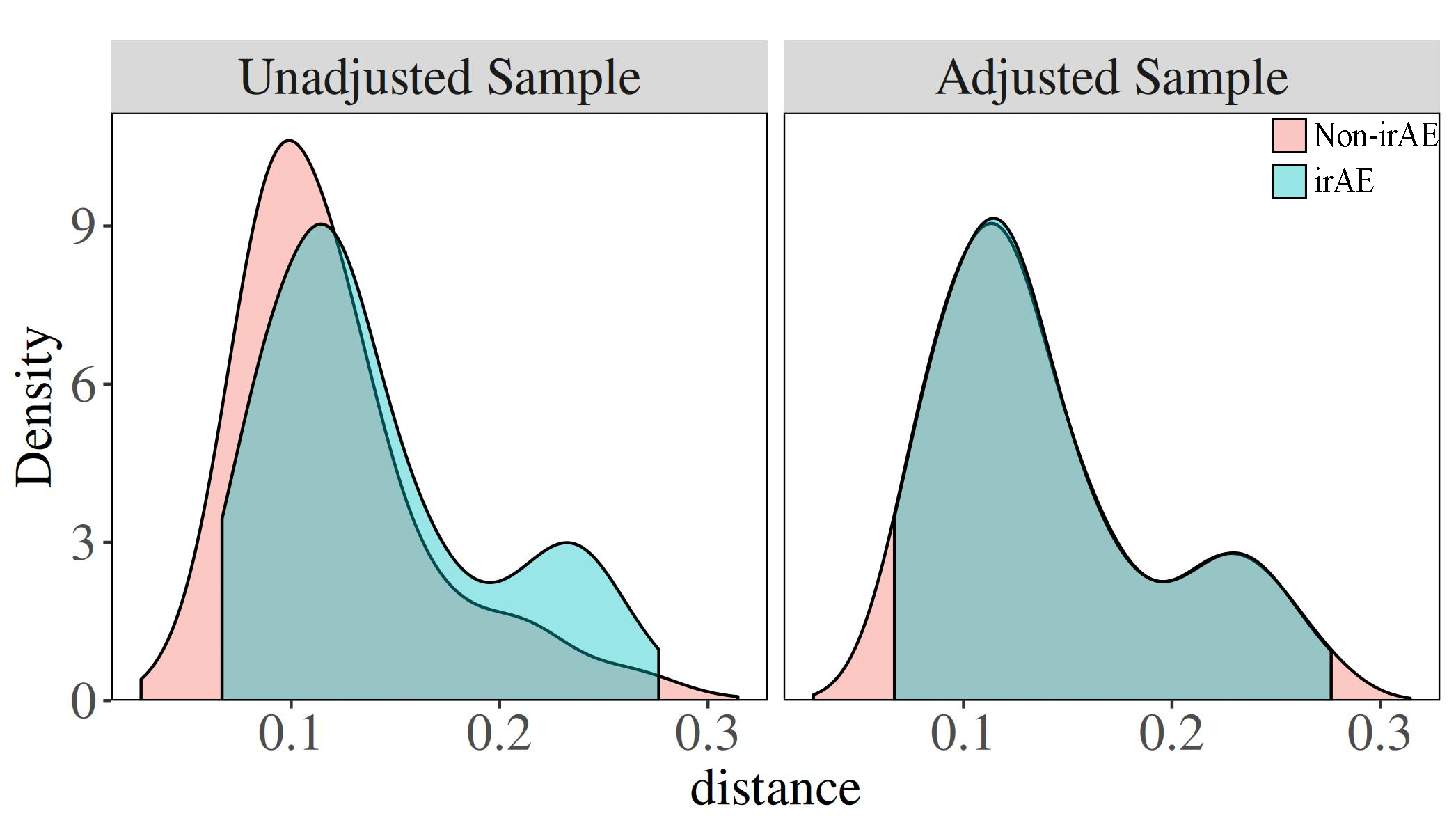** | **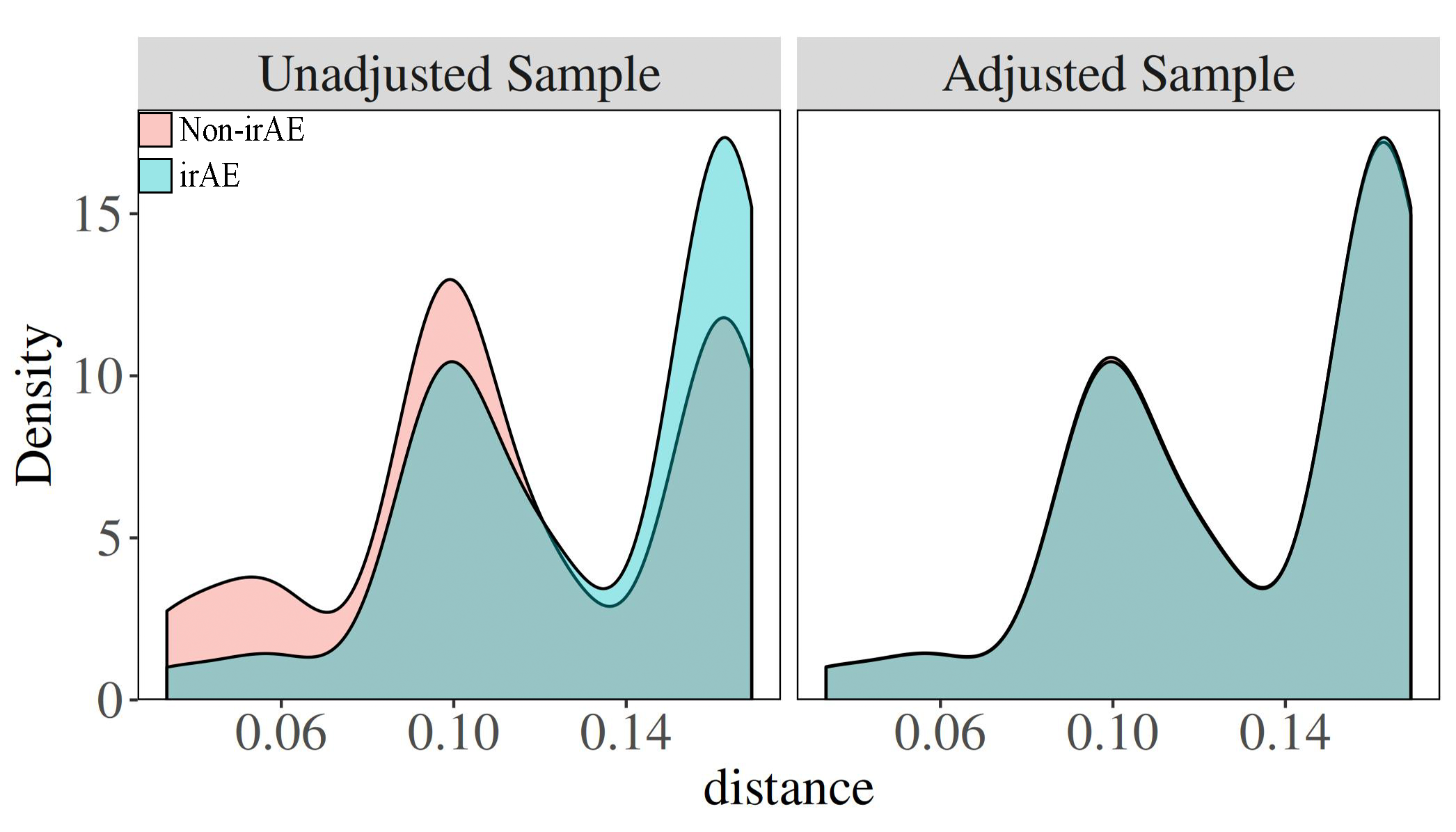** |
| **C** | **D** |
| **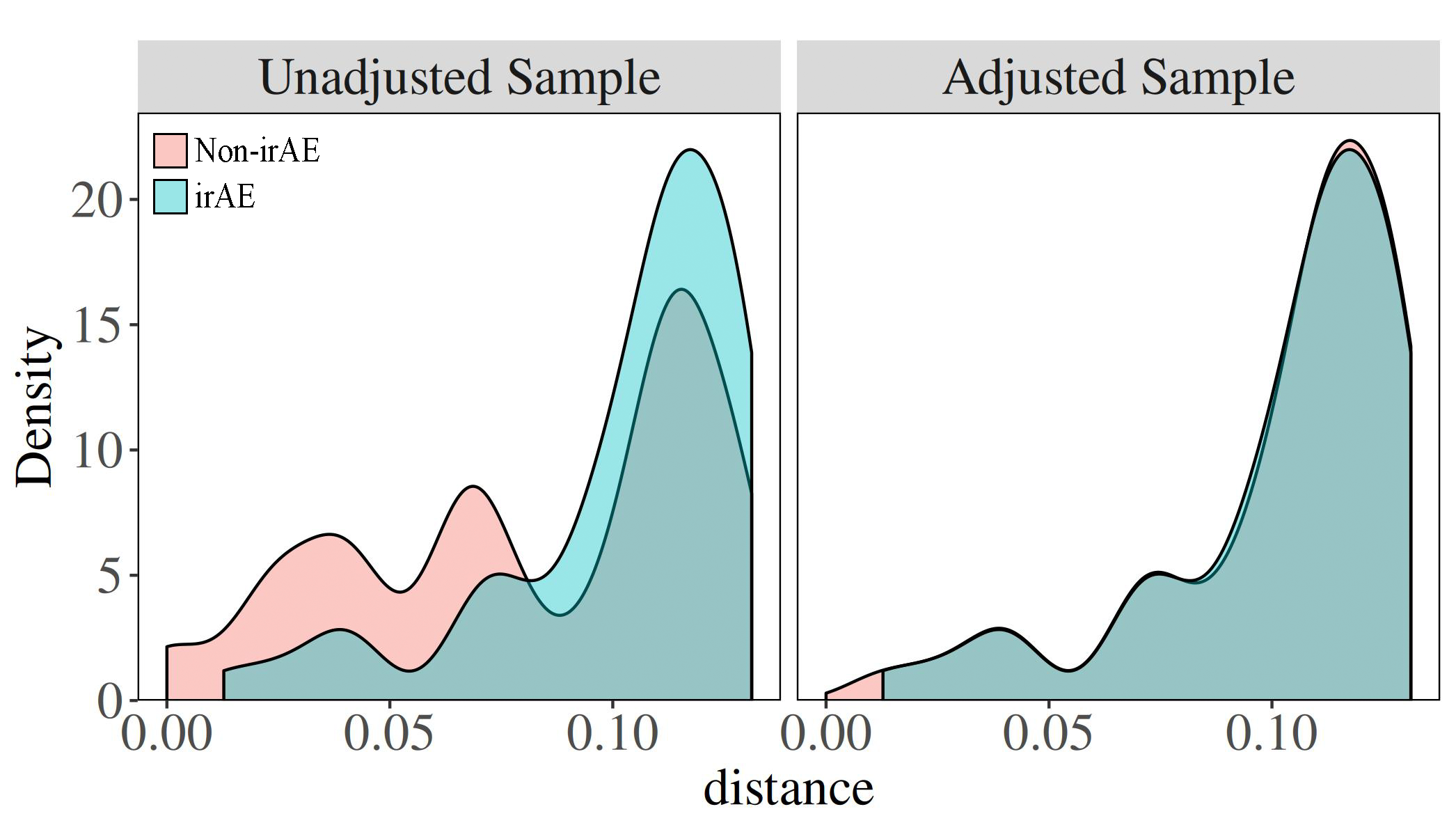** | **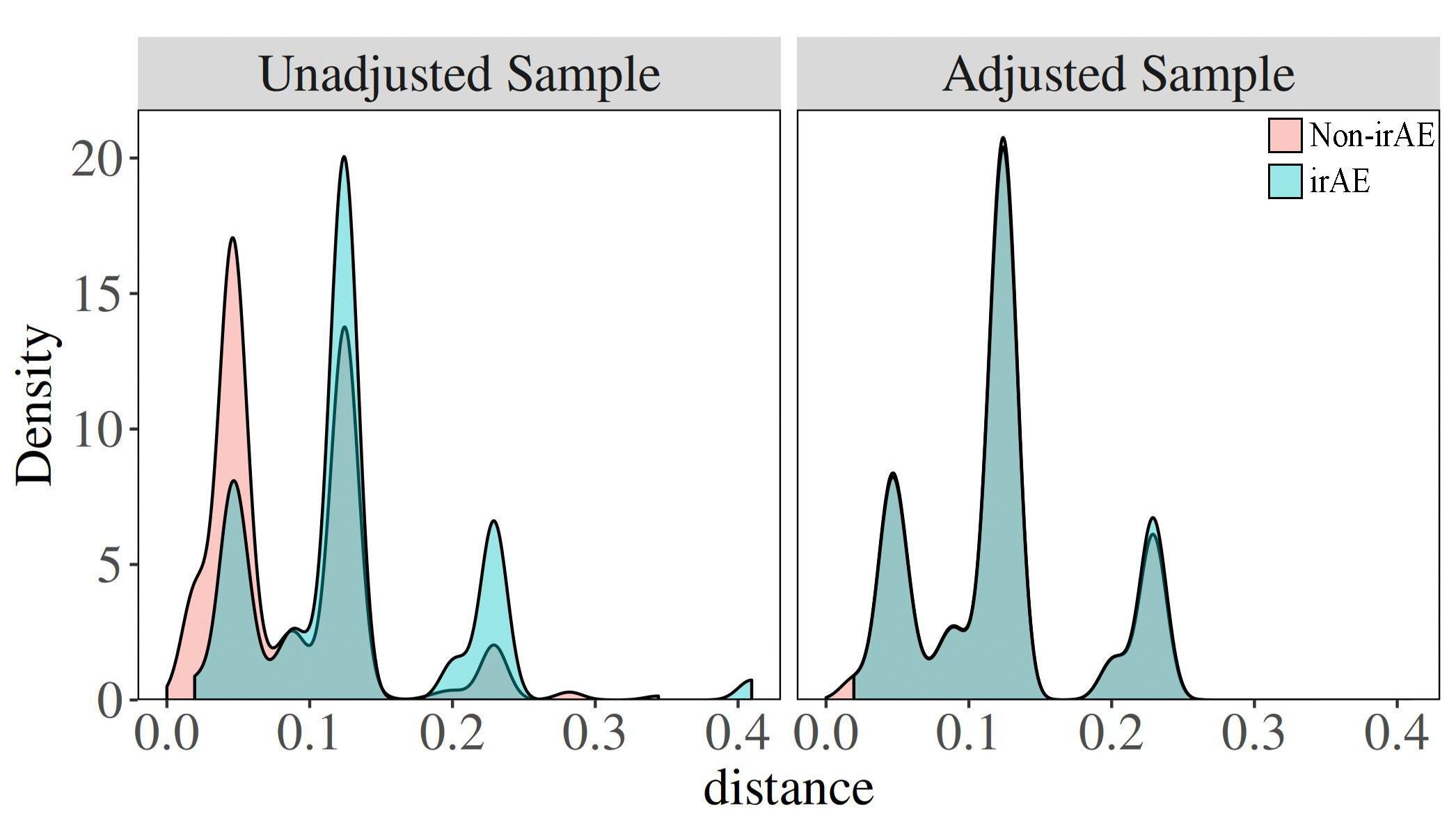** |
| **E** |  |
| **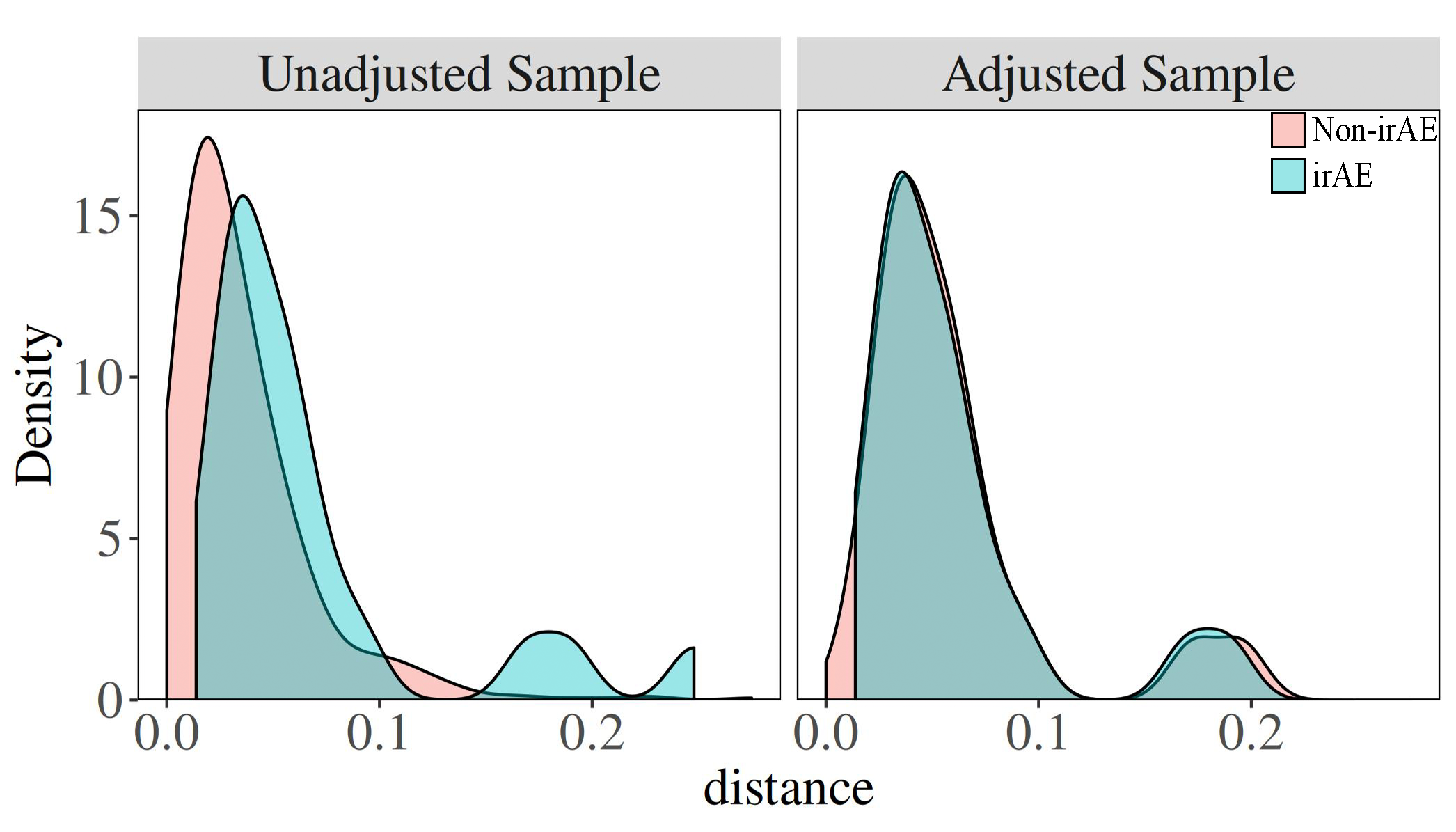** |  |

**Supplementary Figure 1** Comparison of sample distribution balance before and after matching for different toxicity propensity scores (A Endocrine toxicity; B Skin toxicity; C Pulmonary toxicity; D Hepatotoxicity; E Cardiotoxicity)

**Supplementary Table 4** Comparison of baseline characteristics in the propensity score-matched cohort related to endocrine toxicity-associated irAEs

| **Variable** | **Before PSM** | | | | | **After PSM** | | | | |
| --- | --- | --- | --- | --- | --- | --- | --- | --- | --- | --- |
|  | **Non-irAEs** | **irAEs** | **SMD** | **Statistic** | **P** | **Non-irAEs** | **irAEs** | **SMD** | **Statistic** | **P** |
|  | **(n = 588)** | **(n =83 )** |  |  |  | **(n = 160)** | **(n =82 )** |  |  |  |
| **Age, Mean ± SD** | 62.28 ± 9.31 | 64.66 ± 8.90 | 0.268 | t=-2.195 | 0.029 | 64.92 ± 8.31 | 64.57 ± 8.91 | -0.039 | t=0.299 | 0.765 |
| **Gender, n (%)** |  |  |  | χ²=8.891 | 0.003 |  |  |  | χ²=0.433 | 0.511 |
| Female | 120 (20.41) | 29 (34.94) | 0.305 |  |  | 48 (30.00) | 28 (34.15) | 0.087 |  |  |
| Male | 468 (79.59) | 54 (65.06) | -0.305 |  |  | 112 (70.00) | 54 (65.85) | -0.087 |  |  |
| **Cancer, n (%)** |  |  |  | χ²=2.601 | 0.272 |  |  |  | χ²=5.105 | 0.078 |
| Gastrointestinal cancer | 252 (42.86) | 28 (33.73) | -0.193 |  |  | 79 (49.38) | 28 (34.15) | -0.321 |  |  |
| Lung cance | 263 (44.73) | 42 (50.60) | 0.117 |  |  | 62 (38.75) | 41 (50.00) | 0.225 |  |  |
| Other cancer | 73 (12.41) | 13 (15.66) | 0.089 |  |  | 19 (11.88) | 13 (15.85) | 0.109 |  |  |
| **Coronaryheart disease, n (%)** | |  |  | χ²=1.697 | 0.193 |  |  |  | χ²=0.005 | 0.942 |
| No | 542 (92.18) | 73 (87.95) | -0.130 |  |  | 141 (88.12) | 72 (87.80) | -0.010 |  |  |
| Yes | 46 (7.82) | 10 (12.05) | 0.130 |  |  | 19 (11.88) | 10 (12.20) | 0.010 |  |  |
| **Diabete smellitus, n (%)** | |  |  | χ²=0.042 | 0.838 |  |  |  | χ²=0.195 | 0.659 |
| No | 494 (84.01) | 69 (83.13) | -0.024 |  |  | 131 (81.88) | 69 (84.15) | 0.062 |  |  |
| Yes | 94 (15.99) | 14 (16.87) | 0.024 |  |  | 29 (18.12) | 13 (15.85) | -0.062 |  |  |
| **Hypertension, n (%)** | |  |  | χ²=1.410 | 0.235 |  |  |  | χ²=0.003 | 0.958 |
| No | 393 (66.84) | 50 (60.24) | -0.135 |  |  | 97 (60.62) | 50 (60.98) | 0.007 |  |  |
| Yes | 195 (33.16) | 33 (39.76) | 0.135 |  |  | 63 (39.38) | 32 (39.02) | -0.007 |  |  |
| **Smoking, n (%)** |  |  |  | χ²=1.331 | 0.249 |  |  |  | χ²=0.129 | 0.719 |
| No | 272 (46.26) | 44 (53.01) | 0.135 |  |  | 80 (50.00) | 43 (52.44) | 0.049 |  |  |
| Yes | 316 (53.74) | 39 (46.99) | -0.135 |  |  | 80 (50.00) | 39 (47.56) | -0.049 |  |  |
| **Drinking, n (%)** |  |  |  | χ²=0.168 | 0.682 |  |  |  | χ²=0.061 | 0.805 |
| No | 326 (55.44) | 48 (57.83) | 0.048 |  |  | 91 (56.88) | 48 (58.54) | 0.034 |  |  |
| Yes | 262 (44.56) | 35 (42.17) | -0.048 |  |  | 69 (43.12) | 34 (41.46) | -0.034 |  |  |
| **ECOG PS, n (%)** |  |  |  | - | 0.146 |  |  |  | - | 0.223 |
| 0 | 71 (12.07) | 10 (12.05) | -0.001 |  |  | 21 (13.12) | 9 (10.98) | -0.069 |  |  |
| 1 | 493 (83.84) | 65 (78.31) | -0.134 |  |  | 132 (82.50) | 65 (79.27) | -0.080 |  |  |
| 2 | 23 (3.91) | 8 (9.64) | 0.194 |  |  | 6 (3.75) | 8 (9.76) | 0.202 |  |  |
| 3 | 1 (0.17) | 0 (0.00) | -0.044 |  |  | 1 (0.62) | 0 (0.00) | -0.098 |  |  |
| **Metastatic site, n (%)** | |  |  | χ²=1.264 | 0.261 |  |  |  | χ²=2.066 | 0.151 |
| <2 | 330 (56.12) | 52 (62.65) | 0.135 |  |  | 84 (52.50) | 51 (62.20) | 0.200 |  |  |
| ⩾2 | 258 (43.88) | 31 (37.35) | -0.135 |  |  | 76 (47.50) | 31 (37.80) | -0.200 |  |  |
| **Treatment line, n (%)** | |  |  | χ²=2.898 | 0.235 |  |  |  | χ²=4.388 | 0.111 |
| First line | 466 (79.25) | 67 (80.72) | 0.037 |  |  | 119 (74.38) | 66 (80.49) | 0.154 |  |  |
| Second line | 99 (16.84) | 10 (12.05) | -0.147 |  |  | 35 (21.88) | 10 (12.20) | -0.296 |  |  |
| Third line and above | 23 (3.91) | 6 (7.23) | 0.128 |  |  | 6 (3.75) | 6 (7.32) | 0.137 |  |  |
| **Chemotherapy, n (%)** | |  |  | χ²=1.773 | 0.183 |  |  |  | χ²=2.224 | 0.136 |
| No | 63 (10.71) | 13 (15.66) | 0.136 |  |  | 15 (9.38) | 13 (15.85) | 0.177 |  |  |
| Yes | 525 (89.29) | 70 (84.34) | -0.136 |  |  | 145 (90.62) | 69 (84.15) | -0.177 |  |  |
| t: t-test, χ²: Chi-square test, -: Fisher exact, SD: standard deviation | | | | | | | | | | |

**Supplementary Table 5** Comparison of baseline characteristics in the propensity score-matched cohort related to skin toxicity-associated irAEs

| **Variable** | **Before PSM** | | | | | **After PSM** | | | | |
| --- | --- | --- | --- | --- | --- | --- | --- | --- | --- | --- |
|  | **Non-irAEs** | **irAEs** | **SMD** | **Statistic** | **P** | **Non-irAEs** | **irAEs** | **SMD** | **Statistic** | **P** |
|  | **(n = 588)** | **(n =78 )** |  |  |  | **(n = 156)** | **(n =78 )** |  |  |  |
| **Age, Mean ± SD** | 62.28 ± 9.31 | 63.73 ± 9.94 | 0.146 | t=-1.283 | 0.200 | 62.71 ± 9.77 | 63.73 ± 9.94 | 0.103 | t=-0.748 | 0.455 |
| **Gender, n (%)** |  |  |  | χ²=0.603 | 0.437 |  |  |  | χ²=0.000 | 1.000 |
| Female | 120 (20.41) | 13 (16.67) | -0.100 |  |  | 26 (16.67) | 13 (16.67) | 0.000 |  |  |
| Male | 468 (79.59) | 65 (83.33) | 0.100 |  |  | 130 (83.33) | 65 (83.33) | 0.000 |  |  |
| **Cancer, n (%)** |  |  |  | χ²=4.015 | 0.134 |  |  |  | χ²=0.000 | 1.000 |
| Gastrointestinal cancer | 252 (42.86) | 25 (32.05) | -0.232 |  |  | 50 (32.05) | 25 (32.05) | 0.000 |  |  |
| Lung cance | 263 (44.73) | 44 (56.41) | 0.236 |  |  | 88 (56.41) | 44 (56.41) | 0.000 |  |  |
| Other cancer | 73 (12.41) | 9 (11.54) | -0.027 |  |  | 18 (11.54) | 9 (11.54) | 0.000 |  |  |
| **Coronaryheart disease, n (%)** | |  |  | χ²=1.255 | 0.263 |  |  |  | χ²=0.942 | 0.332 |
| No | 542 (92.18) | 69 (88.46) | -0.116 |  |  | 144 (92.31) | 69 (88.46) | -0.120 |  |  |
| Yes | 46 (7.82) | 9 (11.54) | 0.116 |  |  | 12 (7.69) | 9 (11.54) | 0.120 |  |  |
| **Diabete smellitus, n (%)** | |  |  | χ²=0.524 | 0.469 |  |  |  | χ²=0.020 | 0.888 |
| No | 494 (84.01) | 68 (87.18) | 0.095 |  |  | 137 (87.82) | 68 (87.18) | -0.019 |  |  |
| Yes | 94 (15.99) | 10 (12.82) | -0.095 |  |  | 19 (12.18) | 10 (12.82) | 0.019 |  |  |
| **Hypertension, n (%)** | |  |  | χ²=0.038 | 0.844 |  |  |  | χ²=0.153 | 0.696 |
| No | 393 (66.84) | 53 (67.95) | 0.024 |  |  | 102 (65.38) | 53 (67.95) | 0.055 |  |  |
| Yes | 195 (33.16) | 25 (32.05) | -0.024 |  |  | 54 (34.62) | 25 (32.05) | -0.055 |  |  |
| **Smoking, n (%)** |  |  |  | χ²=0.000 | 0.986 |  |  |  | χ²=2.293 | 0.130 |
| No | 272 (46.26) | 36 (46.15) | -0.002 |  |  | 56 (35.90) | 36 (46.15) | 0.206 |  |  |
| Yes | 316 (53.74) | 42 (53.85) | 0.002 |  |  | 100 (64.10) | 42 (53.85) | -0.206 |  |  |
| **Drinking, n (%)** |  |  |  | χ²=0.231 | 0.631 |  |  |  | χ²=0.308 | 0.579 |
| No | 326 (55.44) | 41 (52.56) | -0.058 |  |  | 76 (48.72) | 41 (52.56) | 0.077 |  |  |
| Yes | 262 (44.56) | 37 (47.44) | 0.058 |  |  | 80 (51.28) | 37 (47.44) | -0.077 |  |  |
| **ECOG PS, n (%)** |  |  |  | - | 0.249 |  |  |  | - | 0.056 |
| 0 | 71 (12.07) | 9 (11.54) | -0.017 |  |  | 15 (9.62) | 9 (11.54) | 0.060 |  |  |
| 1 | 493 (83.84) | 62 (79.49) | -0.108 |  |  | 137 (87.82) | 62 (79.49) | -0.206 |  |  |
| 2 | 23 (3.91) | 7 (8.97) | 0.177 |  |  | 3 (1.92) | 7 (8.97) | 0.247 |  |  |
| 3 | 1 (0.17) | 0 (0.00) | -0.044 |  |  | 1 (0.64) | 0 (0.00) | -0.098 |  |  |
| **Metastatic site, n (%)** | |  |  | χ²=1.260 | 0.262 |  |  |  | χ²=0.000 | 1.000 |
| <2 | 330 (56.12) | 49 (62.82) | 0.139 |  |  | 98 (62.82) | 49 (62.82) | 0.000 |  |  |
| ⩾2 | 258 (43.88) | 29 (37.18) | -0.139 |  |  | 58 (37.18) | 29 (37.18) | 0.000 |  |  |
| **Treatment line, n (%)** | |  |  | χ²=5.773 | 0.056 |  |  |  | χ²=0.000 | 1.000 |
| First line | 466 (79.25) | 69 (88.46) | 0.288 |  |  | 138 (88.46) | 69 (88.46) | 0.000 |  |  |
| Second line | 99 (16.84) | 5 (6.41) | -0.426 |  |  | 10 (6.41) | 5 (6.41) | 0.000 |  |  |
| Third line and above | 23 (3.91) | 4 (5.13) | 0.055 |  |  | 8 (5.13) | 4 (5.13) | 0.000 |  |  |
| **Chemotherapy, n (%)** | |  |  | χ²=0.800 | 0.371 |  |  |  | χ²=1.433 | 0.231 |
| No | 63 (10.71) | 11 (14.10) | 0.097 |  |  | 14 (8.97) | 11 (14.10) | 0.147 |  |  |
| Yes | 525 (89.29) | 67 (85.90) | -0.097 |  |  | 142 (91.03) | 67 (85.90) | -0.147 |  |  |
| t: t-test, χ²: Chi-square test, -: Fisher exact, SD: standard deviation | | | | | | | | | | |

|  |
| --- |

**Supplementary Table 6** Comparison of baseline characteristics in the propensity score-matched cohort related to pulmonary toxicity-associated irAEs

| **Variable** | **Before PSM** | | | | | **After PSM** | | | | |
| --- | --- | --- | --- | --- | --- | --- | --- | --- | --- | --- |
|  | **Non-irAEs** | **irAEs** | **SMD** | **Statistic** | **P** | **Non-irAEs** | **irAEs** | **SMD** | **Statistic** | **P** |
|  | **(n = 588)** | **(n =54)** |  |  |  | **(n = 108)** | **(n =54 )** |  |  |  |
| **Age, Mean ± SD** | 62.28 ± 9.31 | 63.22 ± 8.88 | 0.106 | t=-0.715 | 0.475 | 61.95 ± 9.99 | 63.22 ± 8.88 | 0.143 | t=-0.790 | 0.431 |
| **Gender, n (%)** |  |  |  | χ²=2.710 | 0.100 |  |  |  | χ²=0.033 | 0.856 |
| Female | 120 (20.41) | 6 (11.11) | -0.296 |  |  | 11 (10.19) | 6 (11.11) | 0.029 |  |  |
| Male | 468 (79.59) | 48 (88.89) | 0.296 |  |  | 97 (89.81) | 48 (88.89) | -0.029 |  |  |
| **Cancer, n (%)** |  |  |  | χ²=2.313 | 0.315 |  |  |  | χ²=0.069 | 0.966 |
| Gastrointestinal cancer | 252 (42.86) | 26 (48.15) | 0.106 |  |  | 52 (48.15) | 26 (48.15) | 0.000 |  |  |
| Lung cance | 263 (44.73) | 25 (46.30) | 0.031 |  |  | 51 (47.22) | 25 (46.30) | -0.019 |  |  |
| Other cancer | 73 (12.41) | 3 (5.56) | -0.299 |  |  | 5 (4.63) | 3 (5.56) | 0.040 |  |  |
| **Coronaryheart disease, n (%)** | |  |  | χ²=0.691 | 0.406 |  |  |  | χ²=0.000 | 1.000 |
| No | 542 (92.18) | 52 (96.30) | 0.218 |  |  | 103 (95.37) | 52 (96.30) | 0.049 |  |  |
| Yes | 46 (7.82) | 2 (3.70) | -0.218 |  |  | 5 (4.63) | 2 (3.70) | -0.049 |  |  |
| **Diabete smellitus, n (%)** | |  |  | χ²=4.195 | 0.041 |  |  |  | χ²=0.000 | 1.000 |
| No | 494 (84.01) | 51 (94.44) | 0.455 |  |  | 102 (94.44) | 51 (94.44) | 0.000 |  |  |
| Yes | 94 (15.99) | 3 (5.56) | -0.455 |  |  | 6 (5.56) | 3 (5.56) | 0.000 |  |  |
| **Hypertension, n (%)** | |  |  | χ²=0.280 | 0.597 |  |  |  | χ²=0.351 | 0.554 |
| No | 393 (66.84) | 38 (70.37) | 0.077 |  |  | 71 (65.74) | 38 (70.37) | 0.101 |  |  |
| Yes | 195 (33.16) | 16 (29.63) | -0.077 |  |  | 37 (34.26) | 16 (29.63) | -0.101 |  |  |
| **Smoking, n (%)** |  |  |  | χ²=2.447 | 0.118 |  |  |  | χ²=0.000 | 1.000 |
| No | 272 (46.26) | 19 (35.19) | -0.232 |  |  | 38 (35.19) | 19 (35.19) | 0.000 |  |  |
| Yes | 316 (53.74) | 35 (64.81) | 0.232 |  |  | 70 (64.81) | 35 (64.81) | 0.000 |  |  |
| **Drinking, n (%)** |  |  |  | χ²=0.000 | 0.987 |  |  |  | χ²=0.309 | 0.578 |
| No | 326 (55.44) | 30 (55.56) | 0.002 |  |  | 55 (50.93) | 30 (55.56) | 0.093 |  |  |
| Yes | 262 (44.56) | 24 (44.44) | -0.002 |  |  | 53 (49.07) | 24 (44.44) | -0.093 |  |  |
| **ECOG PS, n (%)** | |  |  | - | 0.212 |  |  |  | - | 0.577 |
| 0 | 71 (12.07) | 8 (14.81) | 0.077 |  |  | 19 (17.59) | 8 (14.81) | -0.078 |  |  |
| 1 | 493 (83.84) | 44 (81.48) | -0.061 |  |  | 85 (78.70) | 44 (81.48) | 0.072 |  |  |
| 2 | 23 (3.91) | 1 (1.85) | -0.153 |  |  | 4 (3.70) | 1 (1.85) | -0.137 |  |  |
| 3 | 1 (0.17) | 1 (1.85) | 0.125 |  |  | 0 (0.00) | 1 (1.85) | 0.137 |  |  |
| **Metastatic site, n (%)** | |  |  | χ²=0.006 | 0.936 |  |  |  | χ²=2.793 | 0.095 |
| <2 | 330 (56.12) | 30 (55.56) | -0.011 |  |  | 45 (41.67) | 30 (55.56) | 0.280 |  |  |
| ⩾2 | 258 (43.88) | 24 (44.44) | 0.011 |  |  | 63 (58.33) | 24 (44.44) | -0.280 |  |  |
| **Treatment line, n (%)** | |  |  | χ²=2.217 | 0.330 |  |  |  | χ²=0.022 | 0.883 |
| First line | 466 (79.25) | 45 (83.33) | 0.110 |  |  | 89 (82.41) | 45 (83.33) | 0.025 |  |  |
| Second line | 99 (16.84) | 9 (16.67) | -0.005 |  |  | 19 (17.59) | 9 (16.67) | -0.025 |  |  |
| Third line and above | 23 (3.91) | 0 (0.00) | -0.211 |  |  |  |  |  |  |  |
| **Chemotherapy, n (%)** | |  |  | χ²=1.427 | 0.232 |  |  |  | χ²=0.000 | 1.000 |
| No | 63 (10.71) | 3 (5.56) | -0.225 |  |  | 7 (6.48) | 3 (5.56) | -0.040 |  |  |
| Yes | 525 (89.29) | 51 (94.44) | 0.225 |  |  | 101 (93.52) | 51 (94.44) | 0.040 |  |  |
| t: t-test, χ²: Chi-square test, -: Fisher exact, SD: standard deviation | | | | | | | | | | |

**Supplementary Table 7** Comparison of baseline characteristics in the propensity score-matched cohort related to hepatotoxicity-associated irAEs

| **Variable** | **Before PSM** | | | | | **After PSM** | | | | |
| --- | --- | --- | --- | --- | --- | --- | --- | --- | --- | --- |
|  | **Non-irAEs** | **irAEs** | **SMD** | **Statistic** | **P** | **Non-irAEs** | **irAEs** | **SMD** | **Statistic** | **P** |
|  | **(n = 588)** | **(n =58 )** |  |  |  | **(n = 114)** | **(n =57 )** |  |  |  |
| **Age, Mean ± SD** | 62.28 ± 9.31 | 62.69 ± 9.78 | 0.042 | t=-0.319 | 0.750 | 62.07 ± 10.02 | 62.70 ± 9.87 | 0.064 | t=-0.391 | 0.697 |
| **Gender, n (%)** |  |  |  | χ²=0.130 | 0.719 |  |  |  | χ²=0.676 | 0.411 |
| Female | 120 (20.41) | 13 (22.41) | 0.048 |  |  | 20 (17.54) | 13 (22.81) | 0.125 |  |  |
| Male | 468 (79.59) | 45 (77.59) | -0.048 |  |  | 94 (82.46) | 44 (77.19) | -0.125 |  |  |
| **Cancer, n (%)** |  |  |  | χ²=14.530 | <0.001 |  |  |  | χ²=0.000 | 1.000 |
| Gastrointestinal cancer | 252 (42.86) | 40 (68.97) | 0.564 |  |  | 78 (68.42) | 39 (68.42) | 0.000 |  |  |
| Lung cance | 263 (44.73) | 14 (24.14) | -0.481 |  |  | 28 (24.56) | 14 (24.56) | 0.000 |  |  |
| Other cancer | 73 (12.41) | 4 (6.90) | -0.218 |  |  | 8 (7.02) | 4 (7.02) | 0.000 |  |  |
| **Coronaryheart disease, n (%)** | |  |  | χ²=1.739 | 0.187 |  |  |  | χ²=0.000 | 1.000 |
| No | 542 (92.18) | 50 (86.21) | -0.173 |  |  | 98 (85.96) | 49 (85.96) | 0.000 |  |  |
| Yes | 46 (7.82) | 8 (13.79) | 0.173 |  |  | 16 (14.04) | 8 (14.04) | 0.000 |  |  |
| **Diabete smellitus, n (%)** | |  |  | χ²=0.009 | 0.926 |  |  |  | χ²=0.182 | 0.670 |
| No | 494 (84.01) | 49 (84.48) | 0.013 |  |  | 93 (81.58) | 48 (84.21) | 0.072 |  |  |
| Yes | 94 (15.99) | 9 (15.52) | -0.013 |  |  | 21 (18.42) | 9 (15.79) | -0.072 |  |  |
| **Hypertension, n (%)** | |  |  | χ²=0.995 | 0.318 |  |  |  | χ²=0.199 | 0.656 |
| No | 393 (66.84) | 35 (60.34) | -0.133 |  |  | 72 (63.16) | 34 (59.65) | -0.072 |  |  |
| Yes | 195 (33.16) | 23 (39.66) | 0.133 |  |  | 42 (36.84) | 23 (40.35) | 0.072 |  |  |
| **Smoking, n (%)** |  |  |  | χ²=0.297 | 0.586 |  |  |  | χ²=0.424 | 0.515 |
| No | 272 (46.26) | 29 (50.00) | 0.075 |  |  | 64 (56.14) | 29 (50.88) | -0.105 |  |  |
| Yes | 316 (53.74) | 29 (50.00) | -0.075 |  |  | 50 (43.86) | 28 (49.12) | 0.105 |  |  |
| **Drinking, n (%)** |  |  |  | χ²=0.085 | 0.771 |  |  |  | χ²=0.984 | 0.321 |
| No | 326 (55.44) | 31 (53.45) | -0.040 |  |  | 71 (62.28) | 31 (54.39) | -0.159 |  |  |
| Yes | 262 (44.56) | 27 (46.55) | 0.040 |  |  | 43 (37.72) | 26 (45.61) | 0.159 |  |  |
| **ECOG PS, n (%)** |  |  |  | - | 0.063 |  |  |  | χ²=0.000 | 1.000 |
| 0 | 71 (12.07) | 13 (22.41) | 0.248 |  |  | 26 (22.81) | 13 (22.81) | 0.000 |  |  |
| 1 | 493 (83.84) | 41 (70.69) | -0.289 |  |  | 82 (71.93) | 41 (71.93) | 0.000 |  |  |
| 2 | 23 (3.91) | 4 (6.90) | 0.118 |  |  | 6 (5.26) | 3 (5.26) | 0.000 |  |  |
| 3 | 1 (0.17) | 0 (0.00) | -0.043 |  |  |  |  |  |  |  |
| **Metastatic site, n (%)** | |  |  | χ²=0.153 | 0.696 |  |  |  | χ²=3.848 | 0.050 |
| <2 | 330 (56.12) | 31 (53.45) | -0.054 |  |  | 44 (38.60) | 31 (54.39) | 0.317 |  |  |
| ⩾2 | 258 (43.88) | 27 (46.55) | 0.054 |  |  | 70 (61.40) | 26 (45.61) | -0.317 |  |  |
| **Treatment line, n (%)** | |  |  | χ²=4.717 | 0.095 |  |  |  | - | 1.000 |
| First line | 466 (79.25) | 50 (86.21) | 0.202 |  |  | 100 (87.72) | 50 (87.72) | 0.000 |  |  |
| Second line | 99 (16.84) | 4 (6.90) | -0.392 |  |  | 8 (7.02) | 4 (7.02) | 0.000 |  |  |
| Third line and above | 23 (3.91) | 4 (6.90) | 0.118 |  |  | 6 (5.26) | 3 (5.26) | 0.000 |  |  |
| **Chemotherapy, n (%)** | |  |  | χ²=0.828 | 0.363 |  |  |  | χ²=0.000 | 1.000 |
| No | 63 (10.71) | 4 (6.90) | -0.151 |  |  | 9 (7.89) | 4 (7.02) | -0.034 |  |  |
| Yes | 525 (89.29) | 54 (93.10) | 0.151 |  |  | 105 (92.11) | 53 (92.98) | 0.034 |  |  |
| t: t-test, χ²: Chi-square test, -: Fisher exact, SD: standard deviation | | | | | | | | | | |

**Supplementary Table 8** Comparison of baseline characteristics in the propensity score-matched cohort related to cardiotoxicity-related irAEs

| **Variable** | **Before PSM** | | | | | **After PSM** | | | | |
| --- | --- | --- | --- | --- | --- | --- | --- | --- | --- | --- |
|  | **Non-irAEs** | **irAEs** | **SMD** | **Statistic** | **P** | **Non-irAEs** | **irAEs** | **SMD** | **Statistic** | **P** |
|  | **(n = 588)** | **(n =22)** |  |  |  | **(n = 40)** | **(n =21 )** |  |  |  |
| **Age, Mean ± SD** | 62.28 ± 9.31 | 67.18 ± 9.71 | 0.505 | t=-2.421 | 0.016 | 65.60 ± 6.23 | 66.62 ± 9.57 | 0.106 | t=-0.441 | 0.662 |
| **Gender, n (%)** |  |  |  | χ²=0.000 | 1.000 |  |  |  | χ²=0.063 | 0.803 |
| Female | 120 (20.41) | 5 (22.73) | 0.055 |  |  | 7 (17.50) | 5 (23.81) | 0.148 |  |  |
| Male | 468 (79.59) | 17 (77.27) | -0.055 |  |  | 33 (82.50) | 16 (76.19) | -0.148 |  |  |
| **Cancer, n (%)** |  |  |  | χ²=3.735 | 0.155 |  |  |  | - | 0.850 |
| Gastrointestinal cancer | 252 (42.86) | 5 (22.73) | -0.480 |  |  | 9 (22.50) | 5 (23.81) | 0.031 |  |  |
| Lung cance | 263 (44.73) | 14 (63.64) | 0.393 |  |  | 27 (67.50) | 13 (61.90) | -0.115 |  |  |
| Other cancer | 73 (12.41) | 3 (13.64) | 0.036 |  |  | 4 (10.00) | 3 (14.29) | 0.122 |  |  |
| **Coronaryheart disease, n (%)** | |  |  | χ²=0.000 | 1.000 |  |  |  | χ²=0.041 | 0.839 |
| No | 542 (92.18) | 20 (90.91) | -0.044 |  |  | 34 (85.00) | 19 (90.48) | 0.187 |  |  |
| Yes | 46 (7.82) | 2 (9.09) | 0.044 |  |  | 6 (15.00) | 2 (9.52) | -0.187 |  |  |
| **Diabete smellitus, n (%)** | |  |  | χ²=0.000 | 1.000 |  |  |  | χ²=0.040 | 0.841 |
| No | 494 (84.01) | 19 (86.36) | 0.068 |  |  | 32 (80.00) | 18 (85.71) | 0.163 |  |  |
| Yes | 94 (15.99) | 3 (13.64) | -0.068 |  |  | 8 (20.00) | 3 (14.29) | -0.163 |  |  |
| **Hypertension, n (%)** | |  |  | χ²=1.436 | 0.231 |  |  |  | χ²=0.046 | 0.829 |
| No | 393 (66.84) | 12 (54.55) | -0.247 |  |  | 24 (60.00) | 12 (57.14) | -0.058 |  |  |
| Yes | 195 (33.16) | 10 (45.45) | 0.247 |  |  | 16 (40.00) | 9 (42.86) | 0.058 |  |  |
| **Smoking, n (%)** |  |  |  | χ²=0.119 | 0.730 |  |  |  | χ²=1.720 | 0.190 |
| No | 272 (46.26) | 11 (50.00) | 0.075 |  |  | 14 (35.00) | 11 (52.38) | 0.348 |  |  |
| Yes | 316 (53.74) | 11 (50.00) | -0.075 |  |  | 26 (65.00) | 10 (47.62) | -0.348 |  |  |
| **Drinking, n (%)** |  |  |  | χ²=0.855 | 0.355 |  |  |  | χ²=0.000 | 0.993 |
| No | 326 (55.44) | 10 (45.45) | -0.201 |  |  | 19 (47.50) | 10 (47.62) | 0.002 |  |  |
| Yes | 262 (44.56) | 12 (54.55) | 0.201 |  |  | 21 (52.50) | 11 (52.38) | -0.002 |  |  |
| **ECOG PS, n (%)** |  |  |  | - | 0.435 |  |  |  | - | 0.249 |
| 0 | 71 (12.07) | 4 (18.18) | 0.158 |  |  | 3 (7.50) | 4 (19.05) | 0.294 |  |  |
| 1 | 493 (83.84) | 17 (77.27) | -0.157 |  |  | 36 (90.00) | 16 (76.19) | -0.324 |  |  |
| 2 | 23 (3.91) | 1 (4.55) | 0.030 |  |  | 1 (2.50) | 1 (4.76) | 0.106 |  |  |
| 3 | 1 (0.17) | 0 (0.00) | -0.042 |  |  |  |  |  |  |  |
| **Metastatic site, n (%)** | |  |  | χ²=0.021 | 0.884 |  |  |  | χ²=0.120 | 0.730 |
| <2 | 330 (56.12) | 12 (54.55) | -0.032 |  |  | 21 (52.50) | 12 (57.14) | 0.094 |  |  |
| ⩾2 | 258 (43.88) | 10 (45.45) | 0.032 |  |  | 19 (47.50) | 9 (42.86) | -0.094 |  |  |
| **Treatment line, n (%)** | |  |  | - | 0.398 |  |  |  | χ²=0.008 | 0.929 |
| First line | 466 (79.25) | 16 (72.73) | -0.146 |  |  | 29 (72.50) | 15 (71.43) | -0.024 |  |  |
| Second line | 99 (16.84) | 6 (27.27) | 0.234 |  |  | 11 (27.50) | 6 (28.57) | 0.024 |  |  |
| Third line and above | 23 (3.91) | 0 (0.00) | -0.206 |  |  |  |  |  |  |  |
| **Chemotherapy, n (%)** | |  |  | χ²=1.996 | 0.158 |  |  |  | χ²=0.000 | 1.000 |
| No | 63 (10.71) | 5 (22.73) | 0.287 |  |  | 7 (17.50) | 4 (19.05) | 0.039 |  |  |
| Yes | 525 (89.29) | 17 (77.27) | -0.287 |  |  | 33 (82.50) | 17 (80.95) | -0.039 |  |  |
| t: t-test, χ²: Chi-square test, -: Fisher exact, SD: standard deviation | | | | | | | | | | |


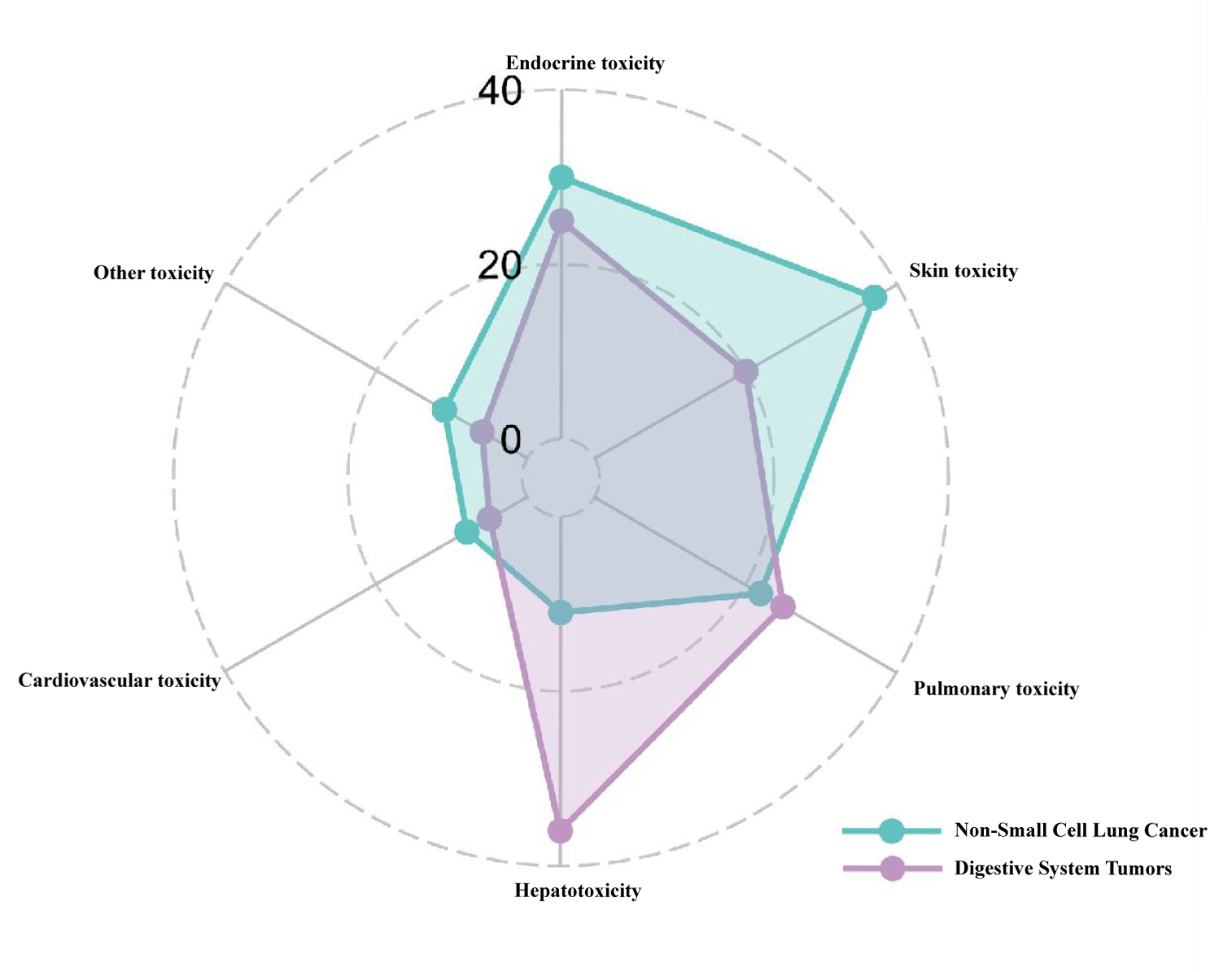


**Supplementary Figure 2** Association network of types and frequency of adverse reactions in patients with lung cancer and digestive tract cancer

| A | B |
| --- | --- |
| 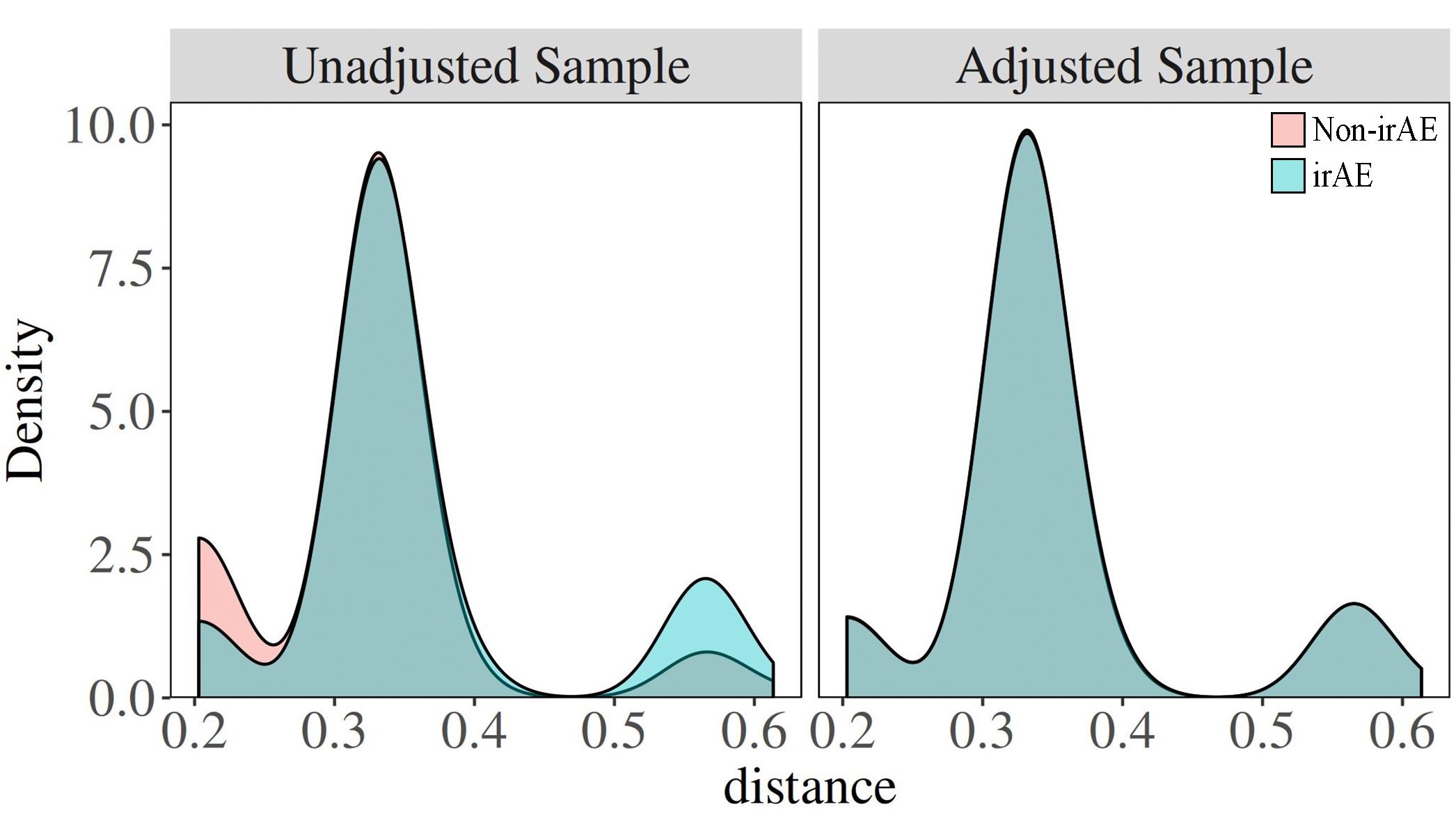 | 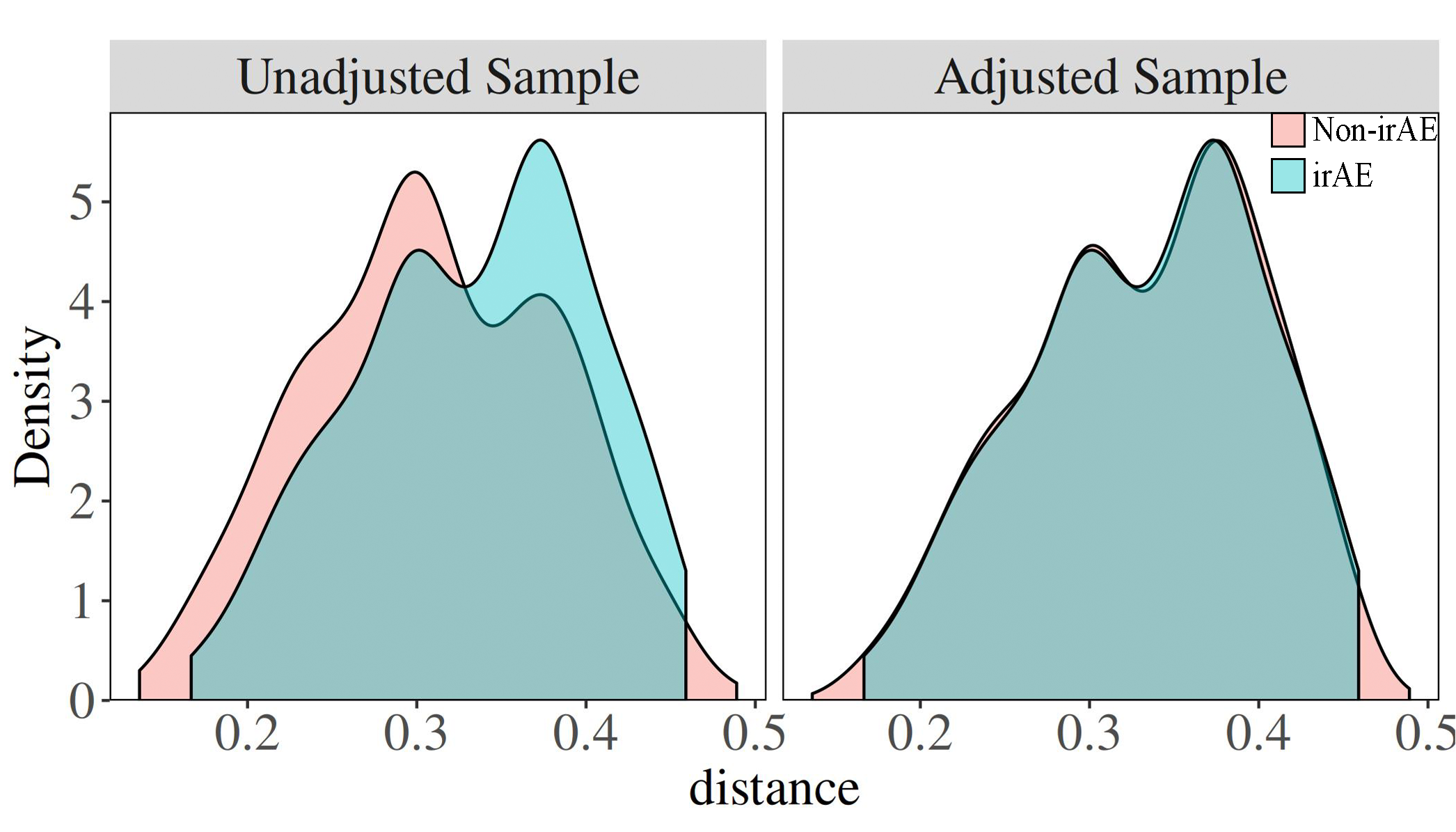 |

**Supplementary Figure 3** Comparison of sample distribution balance before and after propensity score matching for different cancer types (A：Non-small cell lung cancer; B： gastrointestinal cancers)

**Supplementary Table 9** Baseline Patient Characteristics Before and After Propensity Score Matching in thegastrointestinal cancers Subgroup

| **Variable** | **Before PSM** | | | | | **After PSM** | | | | |
| --- | --- | --- | --- | --- | --- | --- | --- | --- | --- | --- |
|  | **Non-irAEs   (n = 263)** | **irAEs  (n = 132)** | **SMD** | **Statistic** | **P** | **Non-irAEs  (n = 232)** | **irAEs  (n = 126)** | **SMD** | **Statistic** | **P** |
| **Age, Mean ± SD** | 63.23 ± 8.47 | 64.08 ± 8.61 | 0.099 | t=-0.942 | 0.347 | 63.37 ± 8.69 | 63.66 ± 8.62 | 0.034 | t=-0.293 | 0.770 |
| **Gender, n (%)** |  |  |  | χ²=0.000 | 0.985 |  |  |  | χ²=0.064 | 0.801 |
| Female | 56 (21.29) | 28 (21.21) | -0.002 |  |  | 44 (20.95) | 27 (22.13) | 0.028 |  |  |
| Male | 207 (78.71) | 104 (78.79) | 0.002 |  |  | 166 (79.05) | 95 (77.87) | -0.028 |  |  |
| **Coronaryheart disease, n (%)** | |  |  | χ²=10.281 | 0.001 |  |  |  | χ²=2.706 | 0.100 |
| No | 246 (93.54) | 110 (83.33) | -0.274 |  |  | 198 (94.29) | 109 (89.34) | -0.160 |  |  |
| Yes | 17 (6.46) | 22 (16.67) | 0.274 |  |  | 12 (5.71) | 13 (10.66) | 0.160 |  |  |
| **Diabete smellitus, n (%)** | |  |  | χ²=0.000 | 0.988 |  |  |  | χ²=0.022 | 0.882 |
| No | 225 (85.55) | 113 (85.61) | 0.002 |  |  | 183 (87.14) | 107 (87.70) | 0.017 |  |  |
| Yes | 38 (14.45) | 19 (14.39) | -0.002 |  |  | 27 (12.86) | 15 (12.30) | -0.017 |  |  |
| **Hypertension, n (%)** |  |  |  | χ²=0.001 | 0.979 |  |  |  | χ²=0.754 | 0.385 |
| No | 171 (65.02) | 86 (65.15) | 0.003 |  |  | 149 (70.95) | 81 (66.39) | -0.097 |  |  |
| Yes | 92 (34.98) | 46 (34.85) | -0.003 |  |  | 61 (29.05) | 41 (33.61) | 0.097 |  |  |
| **Smoking, n (%)** |  |  |  | χ²=1.310 | 0.252 |  |  |  | χ²=0.078 | 0.781 |
| No | 97 (36.88) | 41 (31.06) | -0.126 |  |  | 72 (34.29) | 40 (32.79) | -0.032 |  |  |
| Yes | 166 (63.12) | 91 (68.94) | 0.126 |  |  | 138 (65.71) | 82 (67.21) | 0.032 |  |  |
| **Drinking, n (%)** |  |  |  | χ²=0.673 | 0.412 |  |  |  | χ²=0.291 | 0.590 |
| No | 141 (53.61) | 65 (49.24) | -0.087 |  |  | 108 (51.43) | 59 (48.36) | -0.061 |  |  |
| Yes | 122 (46.39) | 67 (50.76) | 0.087 |  |  | 102 (48.57) | 63 (51.64) | 0.061 |  |  |
| **ECOG PS, n (%)** |  |  |  | χ²=1.331 | 0.514 |  |  |  | χ²=3.406 | 0.182 |
| 0 | 31 (11.79) | 19 (14.39) | 0.074 |  |  | 22 (10.48) | 19 (15.57) | 0.141 |  |  |
| 1 | 221 (84.03) | 105 (79.55) | -0.111 |  |  | 180 (85.71) | 95 (77.87) | -0.189 |  |  |
| 2 | 11 (4.18) | 8 (6.06) | 0.079 |  |  | 8 (3.81) | 8 (6.56) | 0.111 |  |  |
| **Metastatic site, n (%)** |  |  |  | χ²=0.034 | 0.855 |  |  |  | χ²=0.254 | 0.615 |
| <2 | 148 (56.27) | 73 (55.30) | -0.020 |  |  | 123 (58.57) | 68 (55.74) | -0.057 |  |  |
| ⩾2 | 115 (43.73) | 59 (44.70) | 0.020 |  |  | 87 (41.43) | 54 (44.26) | 0.057 |  |  |
| **Treatment line, n (%)** |  |  |  | χ²=6.323 | 0.042 |  |  |  | χ²=0.127 | 0.938 |
| First line | 196 (74.52) | 109 (82.58) | 0.212 |  |  | 175 (83.33) | 100 (81.97) | -0.036 |  |  |
| Second line | 54 (20.53) | 14 (10.61) | -0.322 |  |  | 23 (10.95) | 14 (11.48) | 0.016 |  |  |
| Third line and above | 13 (4.94) | 9 (6.82) | 0.074 |  |  | 12 (5.71) | 8 (6.56) | 0.034 |  |  |
| **Chemotherapy, n (%)** |  |  |  | χ²=2.834 | 0.092 |  |  |  | χ²=0.749 | 0.387 |
| No | 17 (6.46) | 15 (11.36) | 0.154 |  |  | 15 (7.14) | 12 (9.84) | 0.090 |  |  |
| Yes | 246 (93.54) | 117 (88.64) | -0.154 |  |  | 195 (92.86) | 110 (90.16) | -0.090 |  |  |
| t: t-test, χ²: Chi-square test, -: Fisher exact, SD: standard deviation | | | | | | | | | | |

**Supplementary Table 10** Baseline Patient Characteristics Before and After Propensity Score Matching in the gastrointestinal cancers Subgroup

| **Variable** | **Before PSM** | | | | | **After PSM** | | | | |
| --- | --- | --- | --- | --- | --- | --- | --- | --- | --- | --- |
|  | **Non-irAEs   (n = 252)** | **irAEs  (n = 117)** | **SMD** | **Statistic** | **P** | **Non-irAEs  (n = 206)** | **irAEs  (n = 117)** | **SMD** | **Statistic** | **P** |
| **Age, Mean ± SD** | 62.36 ± 9.71 | 64.60 ± 9.18 | 0.244 | t=-2.099 | 0.037 | 62.75 ± 9.52 | 64.32 ± 9.29 | 0.169 | t=-1.394 | 0.164 |
| **Gender, n (%)** |  |  |  | χ²=1.188 | 0.276 |  |  |  | χ²=0.003 | 0.959 |
| Female | 42 (16.67) | 25 (21.37) | 0.115 |  |  | 37 (19.58) | 22 (19.82) | 0.006 |  |  |
| Male | 210 (83.33) | 92 (78.63) | -0.115 |  |  | 152 (80.42) | 89 (80.18) | -0.006 |  |  |
| **Coronaryheart disease, n (%)** | |  |  | χ²=0.544 | 0.461 |  |  |  | χ²=0.004 | 0.949 |
| No | 229 (90.87) | 109 (93.16) | 0.091 |  |  | 175 (92.59) | 103 (92.79) | 0.008 |  |  |
| Yes | 23 (9.13) | 8 (6.84) | -0.091 |  |  | 14 (7.41) | 8 (7.21) | -0.008 |  |  |
| **Diabete smellitus, n (%)** | |  |  | χ²=2.867 | 0.090 |  |  |  | χ²=0.166 | 0.684 |
| No | 204 (80.95) | 103 (88.03) | 0.218 |  |  | 162 (85.71) | 97 (87.39) | 0.050 |  |  |
| Yes | 48 (19.05) | 14 (11.97) | -0.218 |  |  | 27 (14.29) | 14 (12.61) | -0.050 |  |  |
| **Hypertension, n (%)** |  |  |  | χ²=2.273 | 0.132 |  |  |  | χ²=0.671 | 0.413 |
| No | 171 (67.86) | 70 (59.83) | -0.164 |  |  | 123 (65.08) | 67 (60.36) | -0.096 |  |  |
| Yes | 81 (32.14) | 47 (40.17) | 0.164 |  |  | 66 (34.92) | 44 (39.64) | 0.096 |  |  |
| **Smoking, n (%)** |  |  |  | χ²=0.337 | 0.562 |  |  |  | χ²=0.001 | 0.969 |
| No | 134 (53.17) | 66 (56.41) | 0.065 |  |  | 106 (56.08) | 62 (55.86) | -0.005 |  |  |
| Yes | 118 (46.83) | 51 (43.59) | -0.065 |  |  | 83 (43.92) | 49 (44.14) | 0.005 |  |  |
| **Drinking, n (%)** |  |  |  | χ²=0.024 | 0.878 |  |  |  | χ²=0.047 | 0.828 |
| No | 140 (55.56) | 66 (56.41) | 0.017 |  |  | 108 (57.14) | 62 (55.86) | -0.026 |  |  |
| Yes | 112 (44.44) | 51 (43.59) | -0.017 |  |  | 81 (42.86) | 49 (44.14) | 0.026 |  |  |
| **ECOG PS, n (%)** |  |  |  | - | 0.350 |  |  |  | - | 0.954 |
| 0 | 34 (13.49) | 19 (16.24) | 0.074 |  |  | 31 (16.40) | 17 (15.32) | -0.030 |  |  |
| 1 | 212 (84.13) | 92 (78.63) | -0.134 |  |  | 153 (80.95) | 91 (81.98) | 0.027 |  |  |
| 2 | 5 (1.98) | 5 (4.27) | 0.113 |  |  | 4 (2.12) | 3 (2.70) | 0.036 |  |  |
| 3 | 1 (0.40) | 1 (0.85) | 0.050 |  |  | 1 (0.53) | 0 (0.00) | -0.092 |  |  |
| **Metastatic site, n (%)** |  |  |  | χ²=0.456 | 0.499 |  |  |  | χ²=0.355 | 0.551 |
| <2 | 137 (54.37) | 68 (58.12) | 0.076 |  |  | 104 (55.03) | 65 (58.56) | 0.072 |  |  |
| ⩾2 | 115 (45.63) | 49 (41.88) | -0.076 |  |  | 85 (44.97) | 46 (41.44) | -0.072 |  |  |
| **Treatment line, n (%)** |  |  |  | χ²=0.999 | 0.607 |  |  |  | χ²=0.361 | 0.835 |
| First line | 214 (84.92) | 97 (82.91) | -0.054 |  |  | 163 (86.24) | 94 (84.68) | -0.043 |  |  |
| Second line | 32 (12.70) | 15 (12.82) | 0.004 |  |  | 20 (10.58) | 12 (10.81) | 0.007 |  |  |
| Third line and above | 6 (2.38) | 5 (4.27) | 0.094 |  |  | 6 (3.17) | 5 (4.50) | 0.064 |  |  |
| **Chemotherapy, n (%)** |  |  |  | χ²=0.358 | 0.550 |  |  |  | χ²=0.256 | 0.613 |
| No | 17 (6.75) | 6 (5.13) | -0.073 |  |  | 13 (6.88) | 6 (5.41) | -0.065 |  |  |
| Yes | 235 (93.25) | 111 (94.87) | 0.073 |  |  | 176 (93.12) | 105 (94.59) | 0.065 |  |  |
| t: t-test, χ²: Chi-square test, -: Fisher exact, SD: standard deviation | | | | | | | | | | |

**Supplementary Table 11** Association of Immune-Related Adverse Events as a Time-Dependent Covariate with Survival in Non-Small Cell Lung Cancer, by irAE Type

|  | **Univariate analysis** | | **Multivariate analysis^b^** | |
| --- | --- | --- | --- | --- |
|  | **HR(95%CI)** | **p-value** | **HR(95%CI)** | **p-value** |
| **OS** |  |  |  |  |
| irAEs^a^ | 0.984(0.949,1.020) | 0.381 | 0.982(0.944,1.021) | 0.359 |
| Pulmonary toxicity^a^ | 0.965(0.891,1.046) | 0.389 | 1.003(0.959,1.050) | 0.415 |
| Hepatotoxicity^a^ | 1.010(0.937,1.089) | 0.795 | 0.999(0.925,1.078) | 0.977 |
| Endocrine toxicity^a^ | 0.916(0.815,1.029) | 0.140 | 1.197(0.382,3.751) | 0.758 |
| Skin toxicity^a^ | 0.984(0.937,1.033) | 0.506 | 0.967(0.919,1.016) | 0.186 |
| Cardiotoxicity^a^ | 1.035(0.941,1.138) | 0.478 | 1.039(0.469,4.615) | 0.508 |
| **PFS** |  |  |  |  |
| irAEs^a^ | 1.008(0.981,1.035) | 0.584 | 1.009(0.982,1.038) | 0.513 |
| Pulmonary toxicity^a^ | 0.992(0.950,1.035) | 0.707 | 1.424(0.464,4.369) | 0.536 |
| Hepatotoxicity^a^ | 1.073(0.991,1.162) | 0.083 | 1.039(0.957,1.128) | 0.365 |
| Endocrine toxicity^a^ | 1.015(0.979,1.053) | 0.427 | 0.827(0.473,1.445) | 0.505 |
| Skin toxicity^a^ | 0.976(0.940,1.013) | 0.205 | 0.968(0.932,1.005) | 0.093 |
| Cardiotoxicity^a^ | 1.108(0.029,1.192) | 0.006 | 0.877(0.497,1.547) | 0.650 |
| HR: Hazard Ratio, CI: Confidence Interval, OS: Overall Survival, PFS:Progression-Free Survival ^a^Time-dependent covariate. ^b^Multivariate Cox regression analysis. Covariables included demographic factors (gender, age); tumor types (gastrointestinal cancer, lung cancer); comorbidities (coronary heart disease, diabetes mellitus, hypertension); lifestyle factors (smoking, drinking); ECOG performance status; metastatic site; treatment line; and chemotherapy. | | | | |

**Supplementary Table 12** Association of Immune-Related Adverse Events as a Time-Dependent Covariate with Survival in gastrointestinal cancers, by irAE Type

|  | **Univariate analysis** | | **Multivariate analysis^b^** | |
| --- | --- | --- | --- | --- |
|  | **HR(95%CI)** | **p-value** | **HR(95%CI)** | **p-value** |
| **OS** |  |  |  |  |
| irAEs^a^ | 0.959(0.921,0.998) | 0.041 | 0.946(0.907,0.986) | 0.009 |
| Pulmonary toxicity^a^ | 0.798(0.585,1.088) | 0.154 | 0.737(0.362,1.498) | 0.399 |
| Hepatotoxicity^a^ | 0.996(0.954,1.04) | 0.863 | 1.007(0.962,1.053) | 0.771 |
| Endocrine toxicity^a^ | 1.022(0.979,1.066) | 0.326 | 0.693(0.338,1.420) | 0.316 |
| Skin toxicity^a^ | 1.007(0.957,1.061) | 0.785 | 1.022(0.967,1.080) | 0.449 |
| Cardiotoxicity^a^ | 1.011(0.895,1.143) | 0.858 | 0.685(0.335,1.398) | 0.298 |
| **PFS** |  |  |  |  |
| irAEs^a^ | 1.028(0.996,1.06) | 0.085 | 1.028(0.995,1.061) | 0.096 |
| Pulmonary toxicity^a^ | 1.003(0.956,1.052) | 0.909 | 1.147(0.686,1.919) | 0.601 |
| Hepatotoxicity^a^ | 1.020(0.982,1.059) | 0.308 | 1.005(0.954,1.060) | 0.843 |
| Endocrine toxicity^a^ | 0.997(0.95,1.046) | 0.900 | 1.163(0.691,1.958) | 0.569 |
| Skin toxicity^a^ | 1.004(0.953,1.058) | 0.875 | 1.021(0.966,1.080) | 0.462 |
| Cardiotoxicity^a^ | 1.083(0.972,1.207) | 0.150 | 1.112(0.663,1.865) | 0.688 |
| HR: Hazard Ratio, CI: Confidence Interval, OS: Overall Survival, PFS:Progression-Free Survival ^a^Time-dependent covariate. ^b^Multivariate Cox regression analysis. Covariables included demographic factors (gender, age); tumor types (gastrointestinal cancer, lung cancer); comorbidities (coronary heart disease, diabetes mellitus, hypertension); lifestyle factors (smoking, drinking); ECOG performance status; metastatic site; treatment line; and chemotherapy. | | | | |

**Supplementary Table 13** Receiver Operating Characteristic (ROC) curve analysis of inflammatory biomarkers for the prediction of overall survival and determination of the optimal threshold value

|  | **AUC** | **95%CI** | **P value** | **cut of value** | **sensitivity** | **specificity** |
| --- | --- | --- | --- | --- | --- | --- |
| MLR | 0.702 | 0.653-0.751 | ＜0.001 | 0.47 | 0.513 | 0.807 |
| NLR | 0.612 | 0.559-0.665 | ＜0.001 | 3.45 | 0.58 | 0.644 |
| PLR | 0.604 | 0.551-6.57 | ＜0.001 | 186.98 | 0.587 | 0.603 |
| SII | 0.506 | 0.453-0.560 | 0.807 | 888.32 | 0.427 | 0.636 |
| dNLR | 0.601 | 0.549-0.653 | ＜0.001 | 2.49 | 0.507 | 0.701 |
| SIRI | 0.526 | 0.47-0.581 | 0.324 | 2.08 | 0.38 | 0.719 |

**Supplementary Table 14** Univariate and Multivariate Analyses of Blood-Based Indicators Affecting OS and PFS

|  | **OS** | | **PFS** | |
| --- | --- | --- | --- | --- |
| **Variables** | **Univariate Analysis** | **Multivariate Analysis** | **Univariate Analysis** | **Multivariate Analysis** |
|  | **HR (95%CI),P** | **HR (95%CI),P** | **HR (95%CI),P** | **HR (95%CI),P** |
| **MLR ( vs. ≤0.47)** | |  |  |  |
| ＞0.47 | 3.37 (2.49 ~ 4.55), P<0.001 | 3.05 (2.11 ~ 4.41), P<0.001 | 1.51 (1.21 ~ 1.87), P<0.001 | 1.59 (1.22 ~ 2.07), P<0.001 |
| **NLR ( vs. ≤3.45)** | |  |  |  |
| ＞3.45 | 2.24 (1.65 ~ 3.03), P<0.001 | 1.51 (0.87 ~ 2.61), P=0.141 | 1.28 (1.05 ~ 1.56), P=0.015 | 0.95 (0.65 ~ 1.38), P=0.797 |
| **PLR ( vs. ≤186.98)** | |  |  |  |
| ＞186.98 | 2.10 (1.55 ~ 2.84), P<0.001 | 1.75 (1.20 ~ 2.56), P=0.004 | 1.32 (1.09 ~ 1.61), P=0.005 | 1.17 (0.92 ~ 1.50), P=0.204 |
| **dNLR ( vs. ≤2.49)** | |  |  |  |
| ＞2.49 | 2.16 (1.60 ~ 2.92), P<0.001 | 1.41 (0.85 ~ 2.36), P=0.186 | 1.38 (1.12 ~ 1.70), P=0.005 | 1.17 (0.92 ~ 1.50), P=0.111 |
| **SII ( vs. 888.32)** | |  |  |  |
| ＞888.32 | 1.37 (1.01 ~ 1.86), P=0.043 | 0.66 (0.42 ~ 1.04), P=0.072 | 1.20 (0.98 ~ 1.46), P=0.082 | 1.02 (0.76 ~ 1.36), P=0.920 |
| **SIRI ( vs. ≤2.08)** | |  |  |  |
| ＞2.08 | 1.50 (1.10 ~ 2.05), P=0.011 | 0.75 (0.49 ~ 1.16), P=0.195 | 1.12 (0.91 ~ 1.39), P=0.288 | 0.76 (0.56 ~ 1.04), P=0.083 |





**Supplementary Figure 4** Kaplan–Meier survival curves in the clinical cohort stratified by inflammatory markers for (A) PFS for MLR cut-off, (B) PFS for NLR cut-off, (C) PFS for PLR cut-off, (D) PFS for dNLR cut-off, (E) PFS for SII cut-off, (F) PFS for SIRI cut-off. The p values were calculated using the log-rank test (two-sided). CI, confidence interval; NLR,neutrophil-to-lymphocyte ratio; MLR, monocyte-to-lymphocyte ratio; PLR, platelet-to-lymphocyte ratio; dNLR, derived neutrophil-to-lymphocyte ratio; SII, systemic immune-inflammation index; SIRI, systemic inflammation response index; PFS, progression-free survival.

**Supplementary Table 15** Logistic Regression Analysis of Baseline Inflammatory Marker Levels and the Incidence of Immune-Related Adverse Events

| Variables | B | S.E | *P* | OR (95%CI) |
| --- | --- | --- | --- | --- |
|  |  |  |  |  |
| Gender（Male vs Female) | -0.13 | 0.18 | 0.466 | 0.88 (0.61 ~ 1.25) |
| Age | 0.01 | 0.01 | 0.076 | 1.01 (1.00 ~ 1.03) |
| Cancer Type (Gastrointestinal cancer) | |  |  |  |
| Lung cancer | 0.06 | 0.16 | 0.702 | 1.06 (0.78 ~ 1.45) |
| Other cancer | 0.06 | 0.25 | 0.818 | 1.06 (0.65 ~ 1.71) |
| Metastatic site (＞2 vs ≤2） | -0.01 | 0.15 | 0.942 | 0.99 (0.74 ~ 1.33) |
| Treatment line （First line） |  |  |  |  |
| Second line | -0.32 | 0.22 | 0.14 | 0.73 (0.48 ~ 1.11) |
| Third line and above | 0.19 | 0.36 | 0.598 | 1.21 (0.60 ~ 2.45) |
| MLR(＞0.47 vs ≤0.47) | 0.02 | 0.2 | 0.923 | 1.02 (0.69 ~ 1.50) |
| NLR(＞3.45 vs ≤3.45) | -0.17 | 0.26 | 0.512 | 0.84 (0.50 ~ 1.41) |
| PLR(＞186.98 vs ≤186.98) | -0.02 | 0.18 | 0.909 | 0.98 (0.69 ~ 1.39) |
| SII(＞888.32 vs ≤888.32) | 0.06 | 0.21 | 0.763 | 1.07 (0.71 ~ 1.61) |
| dNLR(＞2.49 vs ≤2.49) | -0.18 | 0.25 | 0.479 | 0.84 (0.51 ~ 1.37) |
| SIRI(＞2.08 vs ≤2.08) | 0.22 | 0.22 | 0.318 | 1.24 (0.81 ~ 1.89) |
| CI, confidence interval; NLR,neutrophil-to-lymphocyte ratio; MLR, monocyte-to-lymphocyte ratio; PLR, platelet-to-lymphocyte ratio; dNLR, derived neutrophil-to-lymphocyte ratio; SII, systemic immune-inflammation index; SIRI, systemic inflammation response index; | | | | |


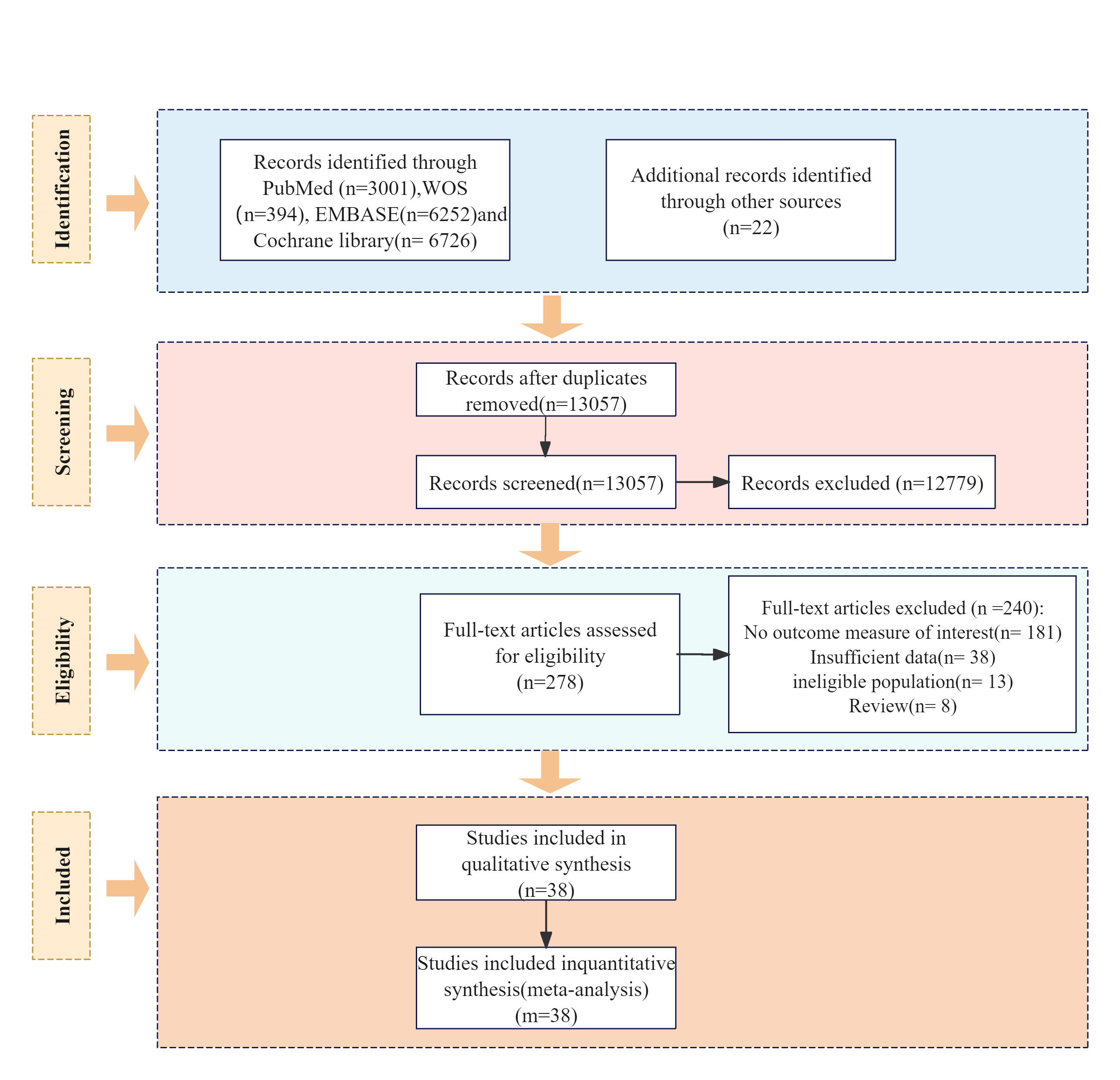


**Supplementary Figure 5** Flow chart of the article identification, inclusion, and exclusion.

**Supplementary Table 16** Assessment of the risk of bias in individual studies through the modified Newcastle-Ottawa Quality Assessment Scale (NOS).

| Author | Study design | Country Area | Sample Size | Gender/Male | Incidence of irAE | NOS |
| --- | --- | --- | --- | --- | --- | --- |
| Beaufils-2023^[1]^ | Retrospective study | France | 1385 | 882（64%） | 90(6.49%) | 9 |
| Foster-2021^[2]^ | Retrospective study | America | 108 | 82（75.9%） | 49(45.40%) | 8 |
| Freeman-Keller-2016^[3]^ | Retrospective study | America | 148 | NA | 101(68.20%) | 9 |
| Kelly-2018^[4]^ | Prospective study | America | 1783 | NA | 295(16.5%) | 9 |
| Morales-Barrera-2023^[5]^ | Retrospective study | Spain | 114 | 95（83%） | 56(49.12%) | 8 |
| Nakano-2020^[6]^ | Retrospective study | Japan | 128 | 70（54.7%） | 61(47.66%) | 8 |
| Verzoni-2019^[7]^ | Retrospective study | Italy | 389 | 291（74.8%） | 124(31.88%) | 8 |
| Wan-2024^[8]^ | Retrospective study | America | 13086 | 7014（53.6%） | 3284(37.7%) | 9 |
| Wan-2024^[8]^ | Retrospective study | America | 26172 | 14051（55.4%） | 5538(30.5%) | 9 |
| Watson-2022^[9]^ | Retrospective study | Canada | 492 | 300（60.9%） | 198(40.24%) | 9 |
| Abu-Sbeih-2019^[10]^ | Retrospective study | America | 2279 | NA | 82(3.60%) | 8 |
| Aleksi -2020^[11]^ | Retrospective study | Canada | 186 | 109（58.6%) | 88(74.32%) | 8 |
| Asdourian-2023^[12]^ | Retrospective study | America | 628 | 371（59.1%） | 312(49.6%) | 7 |
| Bisschop-2019^[13]^ | Prospective study | Netherlands | 147 | 77(52.38%) | 80(55.42%) | 8 |
| Chan-2020^[14]^ | Retrospective study | Australia | 82 | 51（62%） | 33(40.2%) | 8 |
| Cortellini-2019^[15]^ | Retrospective study | Italy | 559 | 379（67.8%） | 231(41.3%) | 8 |
| de la Bruyere-2021^[16]^ | Retrospective study | France | 864 | 603（70%） | 78(9.03%) | 8 |
| Dey-2022^[17]^ | Retrospective study | Multiple | 902 | NA | 215(35%) | 9 |
| Fukushima-2023^[18]^ | Retrospective study | Japan | 150 | 120（80%） | 32(21.33%) | 8 |
| Gulati-2020^[19]^ | Retrospective study | America | 673 | 375（55.7%） | 254(29.16%) | 9 |
| Ikeda-2021^[20]^ | Retrospective study | Japan | 46 | 33（72%) | 33(72%) | 8 |
| Kfoury-2022^[21]^ | Prospective study | France | 577 | 329（57.0%） | 169(27.2%) | 9 |
| Lisberg-2018^[22]^ | Retrospective study | America | 97 | 50（51.5%） | 39(40%) | 9 |
| Maillet-2020^[23]^ | Retrospective study | France | 410 | 295（68%） | 175(40.23%) | 8 |
| Nakamura-2017^[24]^ | Retrospective study | Japan | 37 | 18（48.6%） | 9(25.7%) | 7 |
| Nara-2024^[25]^ | Retrospective study | Japan | 851 | 609（71.6%） | 320(37.6%) | 8 |
| Olsson Ladjevardi-2024^[26]^ | Retrospective study | Sweden | 600 | 348（58%） | 294(49%) | 8 |
| Panagiotou-2024^[27]^ | Retrospective study | Greece | 983 | 738（75.1%） | 513(59.6%) | 8 |
| Ricciuti-2019^[28]^ | Retrospective study | Italy | 195 | 128（65.6%） | 85(43.6%) | 8 |
| Serna‐higuita-2021^[29]^ | Retrospective study | Germany | 319 | 192（60.2%） | 169(52.98%) | 8 |
| Silberg-2022^[30]^ | Retrospective study | Germany | 134 | 94（70.1%） | 85(63.4%) | 8 |
| Wang-2022^[31]^ | Retrospective study | China | 222 | 179（80.6%） | 79(35.6%) | 6 |
| Washino-2023^[32]^ | Retrospective study | Japan | 129 | 92（71%） | 96(74.4%) | 8 |
| Yu-2024^[33]^ | Retrospective study | China | 425 | 332（78.1%） | 127(29.8%) | 8 |
| Zhang-2024^[34]^ | Retrospective study | China | 224 | 198（81.1%） | 140(57%) | 8 |
| Ando-2021^[35]^ | Retrospective study | Japan | 108 | 68（62.9%） | 17(15.7%) | 8 |
| Haratani-2024^[36]^ | Prospective study | Japan | 139 | NA | 81(58%) | 9 |
| Ishihara-2019^[37]^ | Retrospective study | Japan | 47 | 37（78.7%） | 23(48.9%) | 8 |
| Kurokawa-2022^[38]^ | Retrospective study | Japan | 148 | 107（72.3%） | 86(58.11%) | 8 |

[1] Beaufils M, Amodru V, Tejeda M, et al. Dysthyroidism during immune checkpoint inhibitors is associated with improved overall survival in adult cancers: data mining of 1385 electronic patient records [J]. Journal for immunotherapy of cancer, 2023, 11(8). DOI: 10.1136/jitc-2023-006786.

[2] Foster CC, Couey MA, Kochanny SE, et al. Immune-related adverse events are associated with improved response, progression-free survival, and overall survival for patients with head and neck cancer receiving immune checkpoint inhibitors [J]. CANCER, 2021, 127(24): 4565-4573. DOI: 10.1002/cncr.33780.

[3] Freeman-Keller M, Kim Y, Cronin H, et al. Nivolumab in Resected and Unresectable Metastatic Melanoma: Characteristics of Immune-Related Adverse Events and Association with Outcomes [J]. Clinical cancer research : an official journal of the American Association for Cancer Research, 2016, 22(4): 886-894. DOI: 10.1158/1078-0432.Ccr-15-1136.

[4] Kelly K, Manitz J, Patel MR, et al. Association of efficacy and adverse events of special interest of avelumab in the JAVELIN solid tumor and JAVELIN Merkel 200 trials [J]. Journal of Clinical Oncology, 2018, 36(15). DOI: 10.1200/JCO.2018.36.15_suppl.3057.

[5] Morales-Barrera R, Villacampa G, Vidal N, et al. Prevalence of immune-related adverse events and anti-tumor efficacy in advanced/metastatic urothelial carcinoma following immune-checkpoint inhibitor treatment [J]. Clin Transl Oncol, 2023, 25(12): 3556-3564. DOI: 10.1007/s12094-023-03213-6.

[6] Nakano E, Takahashi A, Namikawa K, et al. Correlation between cutaneous adverse events and prognosis in patients with melanoma treated with nivolumab: A single institutional retrospective study [J]. Journal of Dermatology, 2020, 47(6): 622-628. DOI: 10.1111/1346-8138.15309.

[7] Verzoni E, Carteni G, Cortesi E, et al. Real-world efficacy and safety of nivolumab in previously-treated metastatic renal cell carcinoma, and association between immune-related adverse events and survival: the Italian expanded access program [J]. Journal for Immunotherapy of Cancer, 2019, 7. DOI: 10.1186/s40425-019-0579-z.

[8] Wan G, Chen W, Khattab S, et al. Multi-organ immune-related adverse events from immune checkpoint inhibitors and their downstream implications: a retrospective multicohort study [J]. The Lancet Oncology, 2024, 25(8): 1053-1069. DOI: 10.1016/s1470-2045(24)00278-x.

[9] Watson AS, Goutam S, Stukalin I, et al. Association of Immune-Related Adverse Events, Hospitalization, and Therapy Resumption With Survival Among Patients With Metastatic Melanoma Receiving Single-Agent or Combination Immunotherapy [J]. JAMA network open, 2022, 5(12): e2245596. DOI: 10.1001/jamanetworkopen.2022.45596.

[10] Abu-Sbeih H, Tang T, Lu Y, et al. Clinical characteristics and outcomes of immune checkpoint inhibitor-induced pancreatic injury [J]. Journal for ImmunoTherapy of Cancer, 2019, 7(1). DOI: 10.1186/s40425-019-0502-7.

[11] Suo A, Chan Y, Beaulieu C, et al. Anti-PD1-Induced Immune-Related Adverse Events and Survival Outcomes in Advanced Melanoma [J]. The oncologist, 2020, 25(5): 438-446. DOI: 10.1634/theoncologist.2019-0674.

[12] Asdourian MS, Jacoby TV, Shah N, et al. Noncutaneous immune-related adverse events predict overall and progression-free survival in patients with cutaneous toxicities after immune checkpoint inhibitor therapy [J]. Journal of the American Academy of Dermatology, 2023, 88(6): 1368-1370. DOI: 10.1016/j.jaad.2022.12.049.

[13] Bisschop C, Wind TT, Blank CU, et al. Association Between Pembrolizumab-related Adverse Events and Treatment Outcome in Advanced Melanoma: Results From the Dutch Expanded Access Program [J]. Journal of Immunotherapy, 2019, 42(6): 208-214. DOI: 10.1097/cji.0000000000000271.

[14] Chan L, Hwang SJE, Byth K, et al. Survival and prognosis of individuals receiving programmed cell death 1 inhibitor with and without immunologic cutaneous adverse events [J]. Journal of the American Academy of Dermatology, 2020, 82(2): 311-316. DOI: 10.1016/j.jaad.2019.06.035.

[15] Cortellini A, Chiari R, Ricciuti B, et al. Correlations Between the Immune-related Adverse Events Spectrum and Efficacy of Anti-PD1 Immunotherapy in NSCLC Patients [J]. Clinical lung cancer, 2019, 20(4): 237-247.e231. DOI: 10.1016/j.cllc.2019.02.006.

[16] Bruyère CL, Souquet PJ, Dalle S, et al. Investigating the Impact of Immune-Related Adverse Events, Glucocorticoid Use and Immunotherapy Interruption on Long-Term Survival Outcomes. Cancers (Basel). 2021;13(10):2365. Published 2021 May 14. doi:10.3390/cancers13102365.

[17] Dey A, Austin M, Kluger HM, et al. Association between immune-mediated adverse events and efficacy in metastatic non-small-cell lung cancer patients treated with durvalumab and tremelimumab [J]. (1664-3224 (Electronic)). DOI:

[18] Fukushima T, Morimoto M, Kobayashi S, et al. Association Between Immune-Related Adverse Events and Survival in Patients with Hepatocellular Carcinoma Treated With Atezolizumab Plus Bevacizumab [J]. The oncologist, 2023, 28(7): e526-e533. DOI: 10.1093/oncolo/oyad090.

[19] Gulati N, Donnelly D, Qian Y, et al. Revisiting the association between skin toxicity and better response in advanced cancer patients treated with immune checkpoint inhibitors [J]. Journal of translational medicine, 2020, 18(1): 430. DOI: 10.1186/s12967-020-02612-5.

[20] Ikeda T, Ishihara H, Nemoto Y, et al. Prognostic impact of immune-related adverse events in metastatic renal cell carcinoma treated with nivolumab plus ipilimumab [J]. Urologic oncology, 2021, 39(10): 735.e739-735.e716. DOI: 10.1016/j.urolonc.2021.05.012.

[21] Kfoury M, Najean M, Lappara A, et al. Analysis of the association between prospectively collected immune-related adverse events and survival in patients with solid tumor treated with immune-checkpoint blockers, taking into account immortal-time bias [J]. Cancer treatment reviews, 2022, 110: 102452. DOI: 10.1016/j.ctrv.2022.102452.

[22] Lisberg A, Andrew Tucker D, Goldman JW, et al. Treatment-related adverse events predict improved clinical outcome in nsclc patients on KEYNOTE-001 at a single center [J]. Cancer Immunology Research, 2018, 6(3): 288-294. DOI: 10.1158/2326-6066.CIR-17-0063.

[23] Maillet D, Corbaux P, Stelmes J-J, et al. Association between immune-related adverse events and long-term survival outcomes in patients treated with immune checkpoint inhibitors [J]. European Journal of Cancer, 2020, 132: 61-70. DOI: 10.1016/j.ejca.2020.03.017.

[24] Nakamura Y, Tanaka R, Asami Y, et al. Correlation between vitiligo occurrence and clinical benefit in advanced melanoma patients treated with nivolumab: A multi-institutional retrospective study [J]. The Journal of dermatology, 2017, 44(2): 117-122. DOI: 10.1111/1346-8138.13520.

[25] Nara K, Taguchi S, Buti S, et al. Associations of concomitant medications with immune-related adverse events and survival in advanced cancers treated with immune checkpoint inhibitors: a comprehensive pan-cancer analysis [J]. Journal for immunotherapy of cancer, 2024, 12(3). DOI: 10.1136/jitc-2024-008806.

[26] Olsson Ladjevardi C, Koliadi A, Rydén V, et al. Multiple immune-related adverse events secondary to checkpoint inhibitor therapy in patients with advanced cancer: association with treatment effectiveness [J]. Frontiers in Oncology, 2024, 14. DOI: 10.3389/fonc.2024.1399171.

[27] Panagiotou E, Ntouraki S, Vathiotis IA, et al. Endocrine Immune-Related Adverse Events Are Independent Predictors of Survival in Patients with Lung Cancer [J]. Cancers, 2024, 16(9). DOI: 10.3390/cancers16091764.

[28] Ricciuti B, Genova C, De Giglio A, et al. Impact of immune-related adverse events on survival in patients with advanced non-small cell lung cancer treated with nivolumab: long-term outcomes from a multi-institutional analysis [J]. Journal of cancer research and clinical oncology, 2019, 145(2): 479-485. DOI: 10.1007/s00432-018-2805-3.

[29] Serna‐Higuita LM, Amaral T, Forschner A, et al. Association between immune‐related adverse events and survival in 319 stage iv melanoma patients treated with pd‐1‐ based immunotherapy: An approach based on clinical chemistry [J]. Cancers, 2021, 13(23). DOI: 10.3390/cancers13236141.

[30] Silberg M, Krabbe LM, Bögemann M, et al. Immune-Related Adverse Events Can Predict Progression-Free and Overall Survival In Patients With Metastatic Renal Cell Carcinoma Treated With Immune Checkpoint Inhibitors [J]. Clinical genitourinary cancer, 2024, 22(5): 102164. DOI: 10.1016/j.clgc.2024.102164.

[31] Wang W, Gu X, Wang L, et al. The prognostic impact of mild and severe immune-related adverse events in non-small cell lung cancer treated with immune checkpoint inhibitors: a multicenter retrospective study [J]. Cancer immunology, immunotherapy : CII, 2022, 71(7): 1693-1703. DOI: 10.1007/s00262-021-03115-y.

[32] Washino S, Shirotake S, Takeshita H, et al. Association between immune-related adverse events and survival in patients with renal cell carcinoma treated with nivolumab plus ipilimumab: immortal time bias-corrected analysis [J]. International journal of clinical oncology, 2023, 28(12): 1651-1658. DOI: 10.1007/s10147-023-02406-x.

[33] Yu Y, Chen N, Yu S, et al. Association of Immune-Related Adverse Events and the Efficacy of Anti-PD-(L)1 Monotherapy in Non-Small Cell Lung Cancer: Adjusting for Immortal-Time Bias [J]. Cancer research and treatment, 2024, 56(3): 751-764. DOI: 10.4143/crt.2023.1118.

[34] Zhang J, Gao A, Wang S, et al. Correlation between immune-related adverse events and efficacy of PD-(L)1 inhibitors in small cell lung cancer: a multi-center retrospective study [J]. Respiratory research, 2024, 25(1): 256. DOI: 10.1186/s12931-024-02890-3.

[35] Ando T, Ueda A, Ogawa K, et al. Prognosis of Immune-related Adverse Events in Patients With Advanced Gastric Cancer Treated With Nivolumab or Pembrolizumab: A Multicenter Retrospective Analysis [J]. In vivo (Athens, Greece), 2021, 35(1): 475-482. DOI: 10.21873/invivo.12281.

[36] Haratani K, Nakamura A, Mamesaya N, et al. Association of immune-related adverse events with durvalumab efficacy after chemoradiotherapy in patients with unresectable Stage III non-small cell lung cancer [J]. British journal of cancer, 2024, 130(11): 1783-1794. DOI: 10.1038/s41416-024-02662-2.

[37] Ishihara H, Takagi T, Kondo T, et al. Association between immune-related adverse events and prognosis in patients with metastatic renal cell carcinoma treated with nivolumab [J]. Urologic oncology, 2019, 37(6): 355.e321-355.e329. DOI: 10.1016/j.urolonc.2019.03.003.

[38] Kurokawa K, Mitsuishi Y, Shimada N, et al. Association between the efficacy and immune-related adverse events of pembrolizumab and chemotherapy in non-small cell lung cancer patients: a retrospective study [J]. BMC Cancer, 2022, 22(1): 1047. DOI: 10.1186/s12885-022-10133-1.


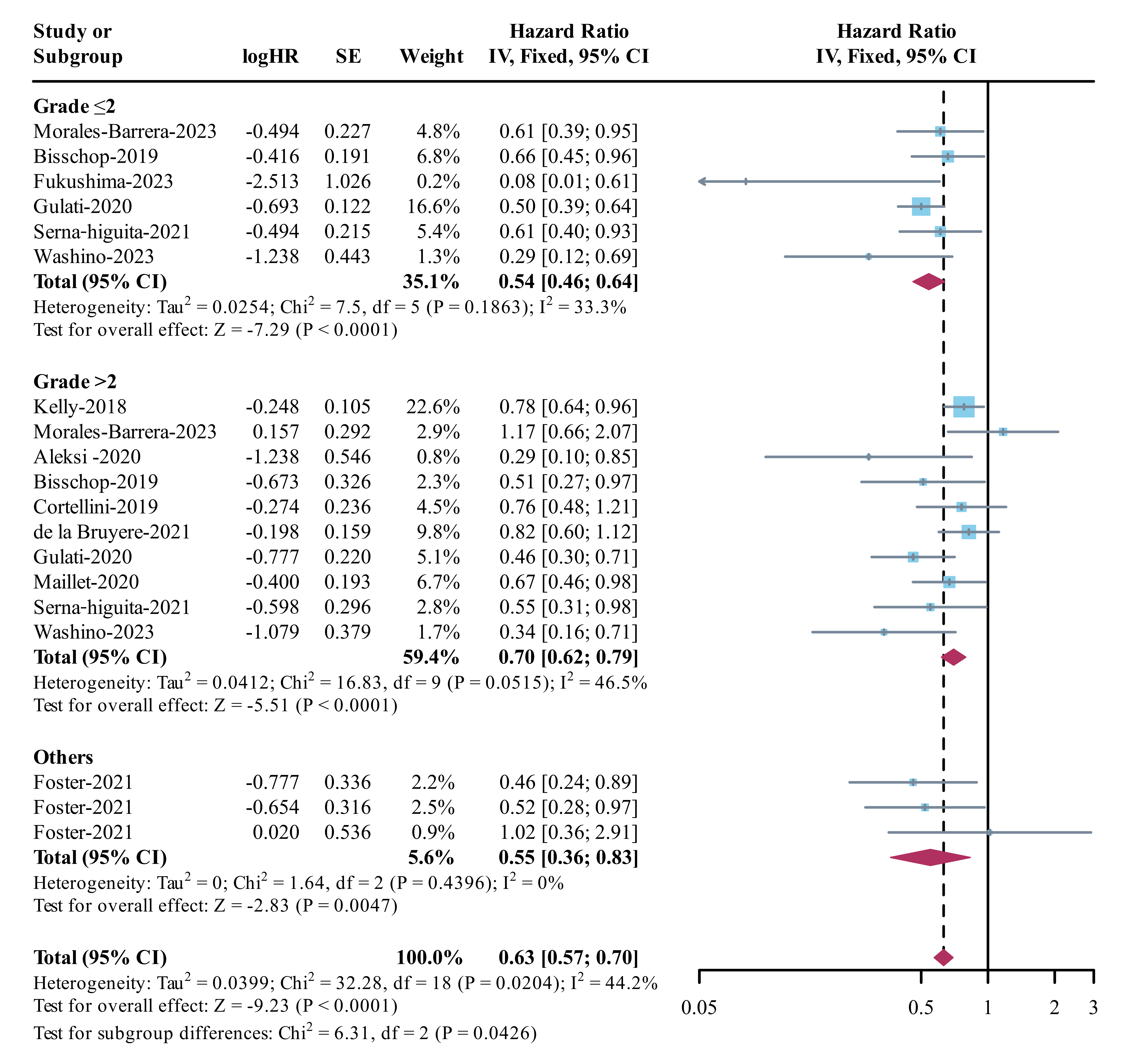


**Supplementary Figure 6. Association of any-grade irAE severity with improved overall survival (OS).**


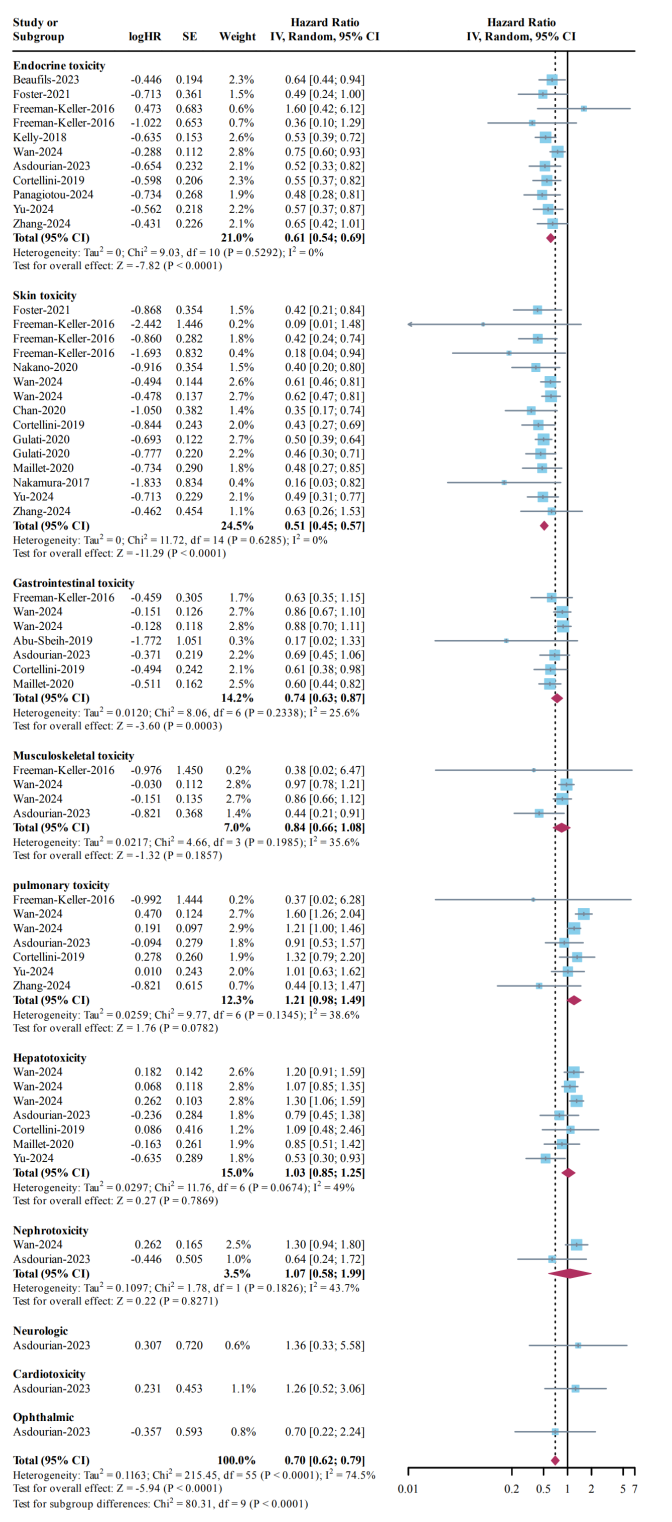


**Supplementary Figure 7. Association of specific irAE types with improved overall survival (OS).**

**
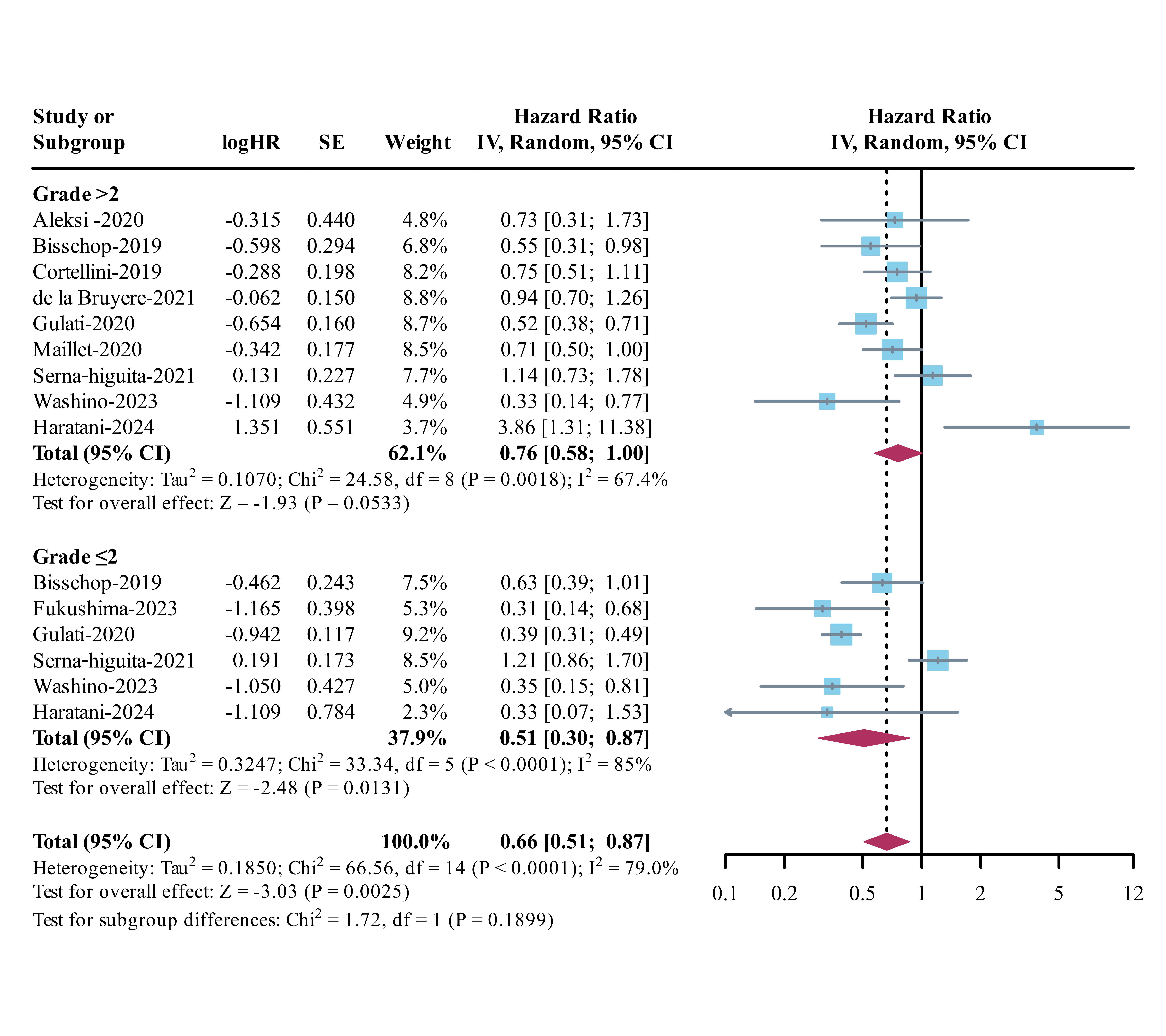
**

**Supplementary Figure 8. Association of any-grade irAE severity with improved progression-free survival (PFS).**


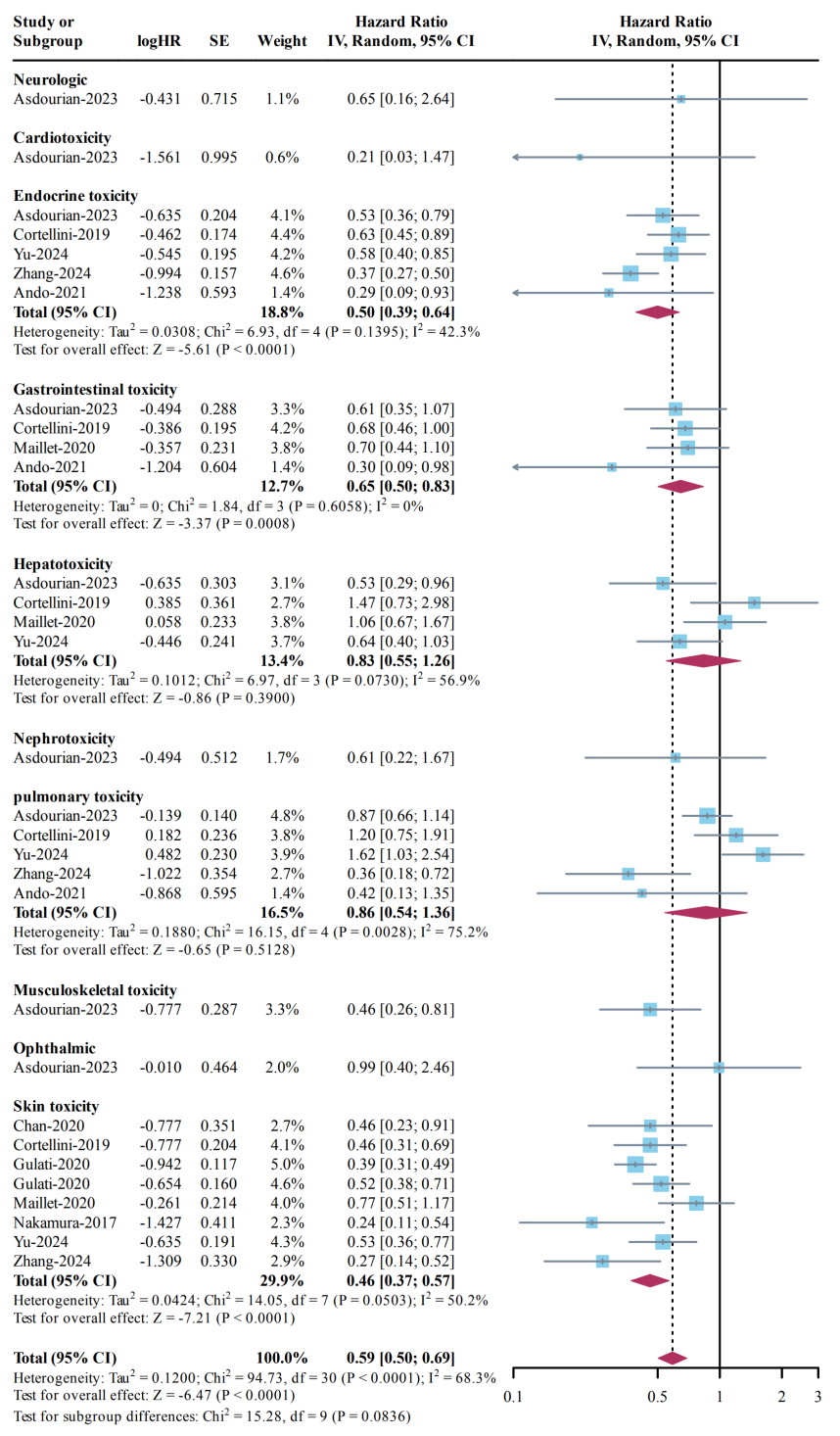


**Supplementary Figure 9. Association of specific irAE types with improved progression-free survival (PFS).**
